# CHMP1B is a target of USP8/UBPY regulated by ubiquitin during endocytosis

**DOI:** 10.1371/journal.pgen.1007456

**Published:** 2018-06-22

**Authors:** Xènia Crespo-Yàñez, Carmen Aguilar-Gurrieri, Anne-Claire Jacomin, Agnès Journet, Magda Mortier, Emmanuel Taillebourg, Emmanuelle Soleilhac, Winfried Weissenhorn, Marie-Odile Fauvarque

**Affiliations:** 1 Institut de Biosciences et Biotechnologies de Grenoble (BIG), Univ. Grenoble Alpes, INSERM U1038, CEA, Grenoble, France; 2 Institut de Biologie Structurale (IBS), Univ. Grenoble Alpes, CNRS, CEA, Grenoble, France; Institut Pasteur, FRANCE

## Abstract

Integration and down-regulation of cell growth and differentiation signals rely on plasma membrane receptor endocytosis and sorting towards either recycling vesicles or degradative lysosomes via multivesicular bodies (MVB). In this process, the endosomal sorting complex-III required for transport (ESCRT-III) controls membrane deformation and scission triggering intraluminal vesicle (ILV) formation at early endosomes. Here, we show that the ESCRT-III member CHMP1B can be ubiquitinated within a flexible loop known to undergo conformational changes during polymerization. We demonstrate further that CHMP1B is deubiquitinated by the ubiquitin specific protease USP8 (syn. UBPY) and found fully devoid of ubiquitin in a ~500 kDa large complex that also contains its ESCRT-III partner IST1. Moreover, EGF stimulation induces the rapid and transient accumulation of ubiquitinated forms of CHMP1B on cell membranes. Accordingly, CHMP1B ubiquitination is necessary for CHMP1B function in both EGF receptor trafficking in human cells and wing development in *Drosophila*. Based on these observations, we propose that CHMP1B is dynamically regulated by ubiquitination in response to EGF and that USP8 triggers CHMP1B deubiquitination possibly favoring its subsequent assembly into a membrane-associated ESCRT-III polymer.

## Author summary

In multicellular organisms, the interpretation and transmission of cell growth and differentiation signals strongly rely on plasma membrane receptors. Once activated by their ligands, these receptors activate downstream signaling cascades and are rapidly internalized into intracellular vesicles that fuse inside the cell to form the endosomal compartment. From there, the receptors are sorted towards either recycling vesicles or degradative lysosomes via multivesicular bodies. Receptors sorting therefore plays a crucial role in the integration and regulation of intracellular signals during development and numerous physio-pathological processes. It requires extensive membrane remodeling and scission events at the level of the endosomal compartment by so-called ESCRT proteins, including CHMP1B. In this study, we provide evidence for dynamic regulation of CHMP1B function and subcellular localization by ubiquitin linkage. We also show the contribution of the ubiquitin specific protease USP8 in this regulation, which is a known actor of intracellular trafficking and Cushing’s disease.

## Introduction

Endocytosis of activated plasma membrane receptors is induced by ubiquitin linkage and plays a crucial role in cell signaling modulation through their subsequent sorting to either recycling vesicles or to lysosomes via multivesicular bodies (MVBs). For example, endocytosis of the Epidermal Growth Factor Receptor (EGFR) represents the major mechanism of long-term attenuation of EGF signaling [1,2]. In this process, the conserved ESCRT (Endosomal Sorting Complex Required for Transport) machinery drives endosomal membrane deformation and scission leading to the formation of intraluminal vesicles (ILVs) within MVBs [3–6]. The ESCRT machinery consists of five complexes called ESCRT-0, I, II, III and VPS4 that are all required for MVB biogenesis: ESCRT-0, I and II cluster the internalized ubiquitinated cargoes and may initiate membrane bending while ESCRT-III and VPS4 are responsible for membrane fission [6–8]. The human ESCRT-III family is composed of 11 proteins termed CHarged Multivesicular Proteins (CHMP1A, B, CHMP2A, B, CHMP3, CHMP4A, B, C, CHMP5, CHMP6 and IST1) which are recruited to membranes [9,10]. They are found in a closed auto-inhibited conformation in the cytosol and activation is thought to displace a C-terminal region from the core helical hairpin [11–15]. Activated ESCRT-III proteins polymerize as homo- or heteromers adopting spiral structures [13,16–21]. CHMP1B polymerization requires extensive conformational changes from the closed conformation to the open polymer conformation. The latter is stabilized by domain swapping of the C-terminal α-helices 4 and 5 and the extension of α-helix 2 by α-helix 3 [22]. Similar conformational changes have been reported for yeast Snf7 (orthologous to CHMP4) indicating common principles for activation and polymerization of the CHMP family of proteins [23,24]. In the particular case of IST1 however, *in vitro* polymerization in a closed conformation has been observed in association with open CHMP1B, resulting in a polymer composed of an external layer of closed IST1 and an internal layer of open CHMP1B [22]. Regulation by phosphorylation has been reported for CHMP1A and CHMP4C [25,26] yet the regulatory mechanisms of ESCRT-III activation and polymerization on membranes *in vivo* remain poorly understood.

The regulatory C-terminal part of ESCRT-III proteins contains one or two MIT interacting motifs (MIM) that recruit partners possessing a Microtubule Interacting and Trafficking (MIT) domain including the Ubiquitin Specific Protease 8 (USP8/UBPY) or the Associated Molecule with SH3 domain of STAM (AMSH) [27–32]. The interaction between the MIT domain of USP8/UBPY and the MIM domain of CHMP1B is required for USP8 association with endosomal membranes and EGFR sorting [30]. In fact, these two Deubiquitinating (DUBs) enzymes are strongly associated with endosomes where they regulate the stability and ubiquitination status of ESCRT-0 members STAM and/or Hrs [30,33–39] as well as of internalized receptors [27,28,30,38–41]. Particularly, both AMSH and USP8/UBPY deubiquitinate the Epidermal Growth Factor Receptor (EGFR), a member of the receptor tyrosine kinase family (RTK), by acting at the level of the plasma membrane and/or of the endosome where deubiquitination of EGFR precedes its incorporation into MVBs [27,33,34,37,41–49]. In addition to EGFR, USP8 deubiquitinates numerous plasma membrane receptors, making this enzyme a promising target in cancer therapy to overcome chemoresistance associated with RTK stabilization [50,51]. Furthermore, gain of function mutations of USP8 have been found in microadenoma of patients with Cushing’s disease, a rare disease where the secretion of large amounts of adenocorticotrophin hormone (ACTH) by pituitary corticotroph adenomas results in excess of glucocorticoids and hypercortisolism putatively due to defective EGFR sorting [52–55].

In the present study, we describe that USP8/UBPY also targets the ESCRT-III machinery. Indeed, we show that CHMP1B is regulated by linkage of ubiquitin and its subsequent removal by USP8/UBPY. The physiological importance of CHMP1B ubiquitination is highlighted by the observation that it is inducible within minutes of EGF stimulation and results in transient accumulation of ubiquitinated forms of CHMP1B on membranes. Moreover, mutation of four lysine residues in, or close to, a flexible loop of CHMP1B makes the protein non-functional in EGFR trafficking in human cells or during *Drosophila* wing morphogenesis.

Thus, our findings establish a new ubiquitin-dependent mechanism controlled by USP8/UBPY (hereafter designed as USP8) that may act as a check-point for the spatial and temporal control of CHMP1B activity such as polymerization at endosomal membranes.

## Results

### Interaction of CHMP1B with USP8 occurs via α-helices 4, 5, and 6 of CHMP1B

**Fig 1:**
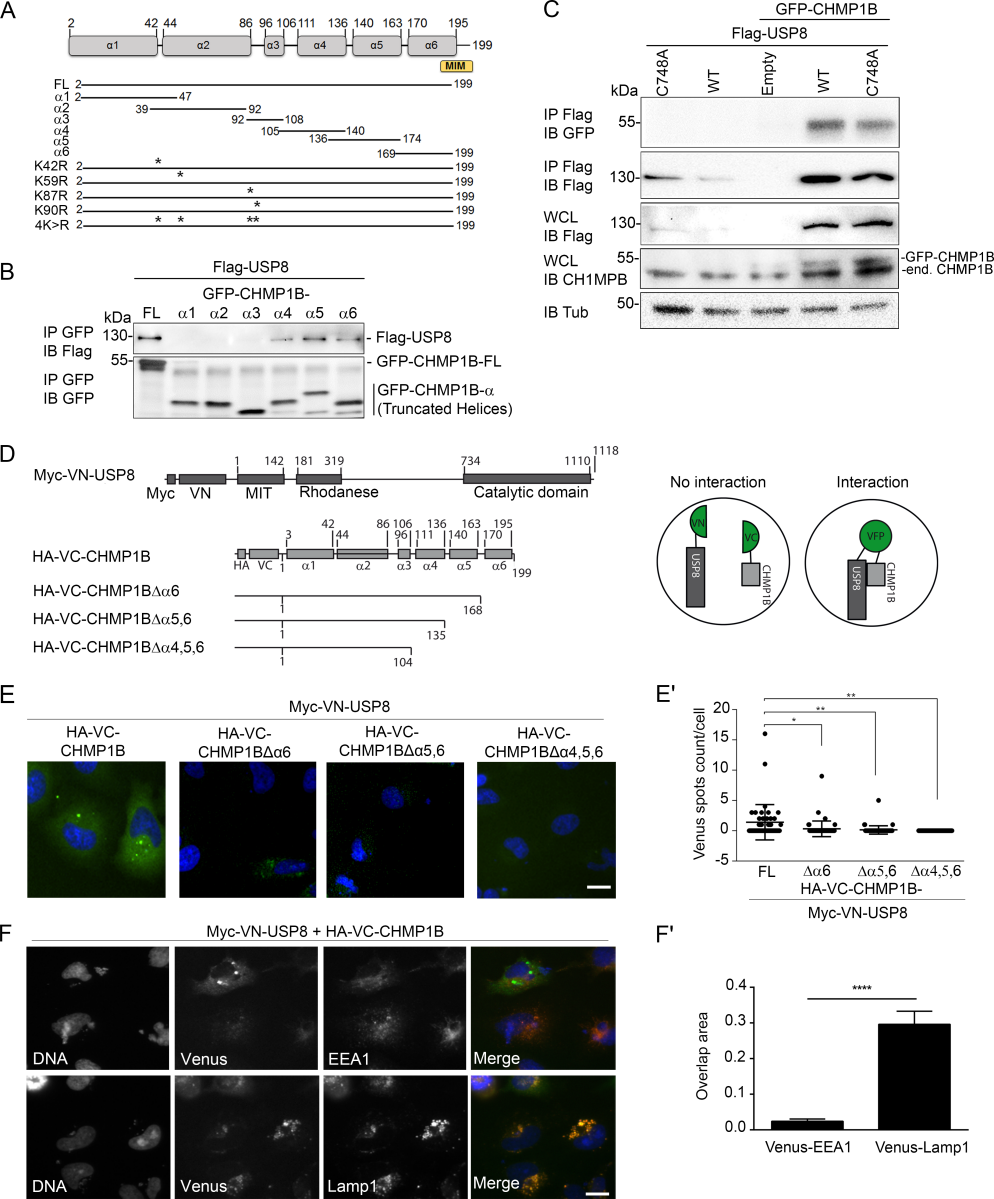
USP8 interacts with helices α4, α5 and α6 of CHMP1B. **A:** Scheme of CHMP1B with six predicted α-helices (α1- α6) and cDNA constructs used in this study. The numbers indicate positions of amino acid residues. Asterisk (*) indicate the lysine residues in positions 42, 59, 87 and 90. **B, C:** HEK293T cells were co-transfected with GFP-CHMP1B and Flag-USP8 constructs, and cell lysates were immunoprecipitated (IP) with anti-GFP or anti-Flag antibodies. IPs were revealed by western blot (IB) using either anti-GFP or anti-Flag antibodies. In (C), whole cell lysates (WCL) were immunoblotted with anti-Flag, anti-CHMP1B or anti-Tub to reveal protein amounts. Note that expression of each protein CHMP1B or USP8 stabilizes the other partner. **D:** Representation of the Venus Fluorescent protein (VFP) fusion constructs carrying Myc or HA tag. VN: Venus Nter, VC: Venus Cter. The numbers indicate positions of amino acid residues in USP8 or CHMP1B. **E:** VFP fluorescence of fixed HeLa cells co-transfected with Myc-VN-USP8 and HA-VC-CHMP1B. Scale bar: 20 μm. In (E’), quantification of VFP spots was performed using HCS Studio software on Myc and HA positive cells only (i.e. in transfected cells). Vertical axis indicates Venus spots count per HA and Myc positive cell. Scatter dot plots represent one representative experiment out of three. Values are mean ± SD. *p<0.01; **p<0.05 (Student’s t-test). **F:** HeLa cells were co-transfected with Myc-VN-USP8 and HA-VC-CHMP1B as in E and immunostained with early (EEA1) or late (LAMP1) endosomes markers. In (F’), quantification of overlap between USP8-CHMP1B VFP spots and EEA1, or LAMP1 is represented as a fraction of total VFP fluorescence. Vertical axis indicates overlap area. Histogram represent one representative experiment out of three. Values are Mean ± S.E.M. ****p<0.0001 (Student’s t-test).

CHMP1B was previously shown to interact with USP8 in both co-immunoprecipitation and yeast two-hybrid experiments [30,56]. In order to further map the domains of CHMP1B implicated in this interaction, GFP-tagged constructs of full length or helical fragments of CHMP1B (Fig 1A) [57] were expressed in HEK293T cells and tested for their ability to co-immunoprecipitate with Flag-USP8. Full-length CHMP1B and α-helices 4, 5 and 6 interacted with Flag-USP8 in this assay (Fig 1B). Interaction of CHMP1B was maintained with a catalytically-inactive version of the enzyme (USP8^C748A^) (Fig 1C). These results map the interacting region of USP8 to CHMP1B residues 105 to 199, similar to the CHMP1B-Spastin interaction [58].

Next, we designed a Venus complementation assay in which USP8 was fused to the N-ter of Venus (VN-USP8) and full length or truncated CHMP1B constructs to its C-ter (VC-CHMP1B) (Fig 1D). The expression of corresponding constructs was verified by immunoblot (S1 Fig). In this assay, a cytoplasmic signal was clearly observed upon co-expression of VN-USP8 and VC-CHMP1B indicating a direct interaction between the two partners *in vivo* (Fig 1E and 1E’). Consistent with the above results, truncation of the helices situated in the C-ter part of CHMP1B led to a decrease (Δα6 and Δα5,6 constructs) or a total loss (Δα4,5,6) of fluorescence (Fig 1E and 1E’). Cells transfected with VN-USP8 and VC-CHMP1B were stained with a set of endosomal markers revealing a strong overlap of the Venus signal with Lamp1, a marker of late endosomes/lysosomes, but not with the early endosomal marker EEA1 (Fig 1F and 1F’).

Taken together, these observations confirm that the two proteins USP8 and CHMP1B are part of a same protein complex in living cells, interacting at the level of the late endosome, which is consistent with the known function of CHMP1B in the multivesicular body biogenesis and sorting of receptors.

### CHMP1B is ubiquitinated within its N-terminal core

**Fig 2:**
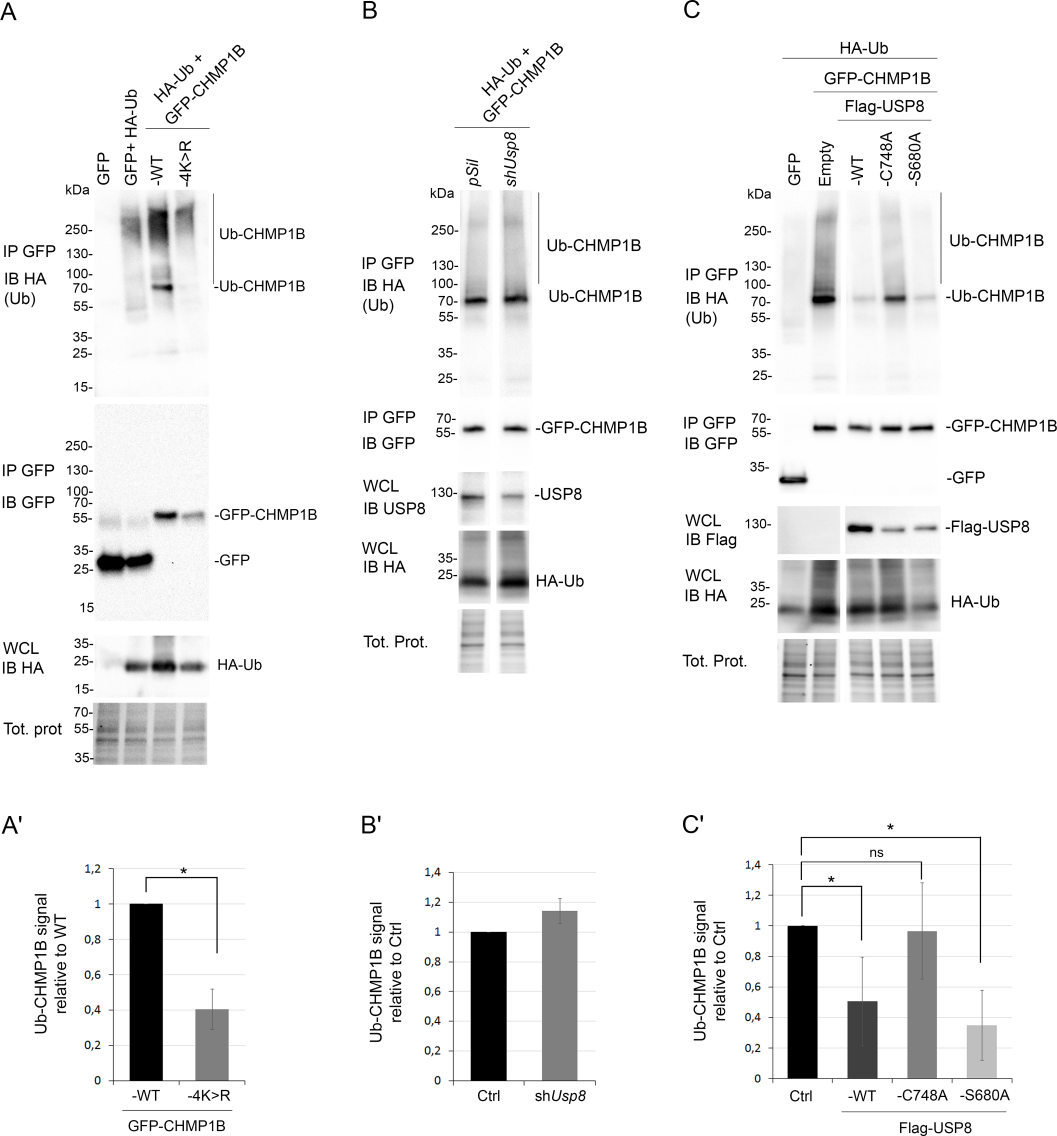
CHMP1B ubiquitination is controlled by USP8. **A-C:** Wild-type or mutated GFP-CHMP1B constructs were transfected into HEK293T cells jointly with HA-ubiquitin (HA-Ub). In (B), cells were co-transfected with sh*Usp8* and in (C), cells were co-transfected with Flag-USP8, Flag-USP8^C748A^ or Flag-USP8^S680A^ constructs. Immunoprecipitations (IP) were carried out with anti-GFP antibodies after strong denaturation of the lysate and proteins were analyzed by immunoblot (IB) using either anti-HA (Ub) or anti-GFP (CHMP1B) antibodies. Whole cell lysates (WCL) were immunoblotted with anti-HA to reveal transfected Ub-HA and, in (B), with anti-USP8 to reveal endogenous USP8, and in (C), with anti-Flag to reveal Flag-USP8 constructs. Total protein amount is shown. **A’, B’, C’:** Quantification by densitometry of blots in A, B or C, respectively. Normalized signals are expressed as a fold-change over CHMP1B wild-type basal ubiquitination levels. Histograms represent the mean of two to three independent experiments. Error bars indicate the range. Values are mean ± SD. *p<0.05 (Student’s t-test).

We next investigated whether CHMP1B is ubiquitinated in cells. To this end, the GFP-CHMP1B protein was expressed in HEK293T cells together with HA-ubiquitin (HA-Ub) [59] and immunoprecipitated from cell lysates with an antibody against GFP. To specifically detect ubiquitin moieties covalently linked to CHMP1B and not to putative partners, highly stringent conditions were used for immunoprecipitation. Western blot of GFP immunoprecipitated product using anti-HA antibodies revealed the presence of a major Ub-CHMP1B product migrating at 70kDa on SDS-PAGE, putatively corresponding to a mono- or di-ubiquitinated form of the recombinant protein, as well as higher molecular weight (*mw*) species that may correspond to multi-mono- or poly-ubiquitinated forms of CHMP1B (Fig 2A, “WT”). Accordingly, previous whole-proteome analyses revealed the existence of ubiquitin linkage at multiple sites of the CHMP1B protein [60–62].

We then generated a CHMP1B mutant construct in which four lysine residues exposed to the solvent were replaced by arginine residues (CHMP1B-4K>R) (Fig 1A, S2A–S2H Fig). This mutant displayed a strong reduction of ubiquitin linkage compared to wild-type CHMP1B (Fig 2A and 2A’; S2I Fig). We also observed a slight reduction of ubiquitin-linked forms of CHMP1B carrying a single K>R substitution in the case of either the K87>R or the K90>R but not in the case of the K42>R or the K59>R single substitutions (S2I Fig). Residual ubiquitination observed with the CHMP1B-4K>R construct may result from other ubiquitination sites within the protein [60–62].

These results indicate that GFP-CHMP1B is ubiquitinated in human cells and that ubiquitin linkage occurs mostly at lysine residues K87 or K90. Remarkably, these two lysine residues are located in the flexible linker between α2 and α3 which becomes helical in the polymer structure (S2A–S2H Fig) [22].

### The ubiquitination status of CHMP1B is controlled by USP8

We then tested whether ubiquitinated CHMP1B is a target of the ubiquitin hydrolase activity of USP8. To this end, HEK293T cells were co-transfected with HA-Ub and GFP-CHMP1B together with *Usp8* silencing (*shUsp8*) or wild-type or mutated USP8 expressing constructs [63]. Silencing of *Usp8* was only partial and resulted in a slight increase of the ubiquitinated pool of GFP-CHMP1B (Fig 2B and 2B’). In contrast, the expression of the wild-type or the constitutively active form USP8^S680A^, but not of the catalytic mutant USP8^C748A^, caused a strong reduction of the Ub-CHMP1B pool (Fig 2C and 2C’). We repeated the experiment using antibodies specifically directed against K48-linked ubiquitin polymers showing that GFP-CHMP1B is unlikely to be modified by this type of chains or at very low level (S2J Fig). Reinforcing this observation, inhibiting the proteasomal activity did neither increase the amount of ubiquitinated GFP-CHMP1B nor stabilize the protein (S2K Fig). In these experiments again, Ub-CHMP1B forms were strongly reduced by expressing USP8 or USP8^S680A^, but not the catalytic mutant USP8^C748A^ (S2J and S2K Fig). Our results thus strongly suggest that USP8 deubiquitinates CHMP1B.

### Endogenous CHMP1B is part of an ESCRT-III complex containing IST1

**Fig 3:**
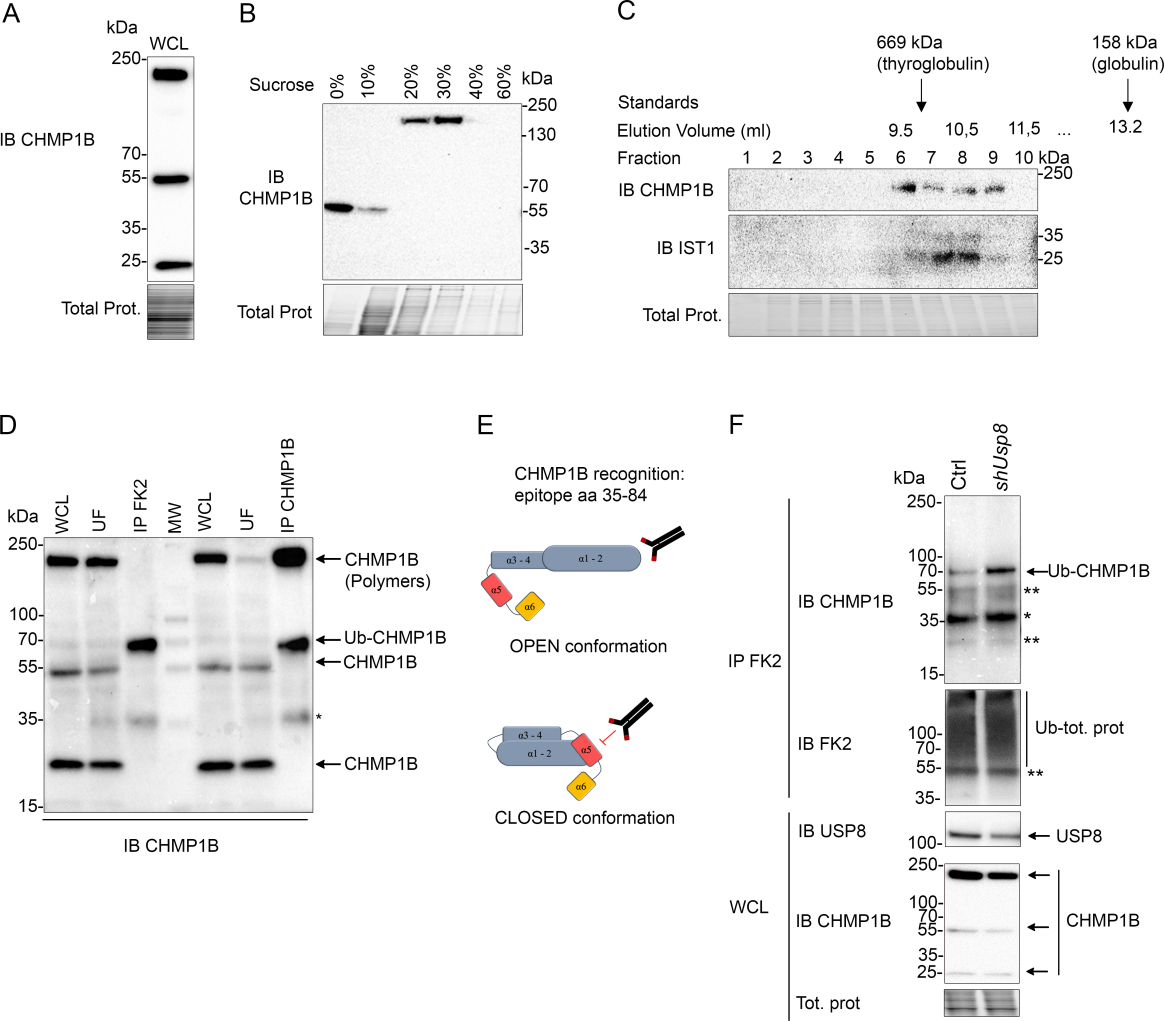
Polymeric CHMP1B belongs to a CHMP1B IST1 complex and is free of ubiquitin. **A:** HEK293T whole cell lysate was analyzed by immunoblot with anti-CHMP1B antibody. **B**: HEK293T whole cell lysates were separated by sucrose gradient centrifugation. The different fractions were separated by SDS-PAGE and CHMP1B was visualized by immunoblot (IB). **C**: The 30% sucrose gradient fraction (in B) was concentrated and separated on a Superdex 200 column. 0.5 ml fractions were analyzed by IB against CHMP1B or IST1. The elution volume of marker proteins are indicated; thyroglobulin, 669 kDa, elutes at 9.8 ml; γ-globulin, 158 kDa, elutes at 13.2 ml. **D:** HEK293T whole cell lysate was immunoprecipitated with the FK2 (left) or the anti-CHMP1B antibody (right) as indicated on the top. The whole cell lysate (WCL), the unbound fraction (UF) and the IPs were analyzed by IB against CHMP1B. Asterisk indicates unspecific band (see S3C Fig). **E:** Schematic representation of the recognition site of the antibody against CHMP1B. **F:** HEK293T cells were transiently transfected with control (pSilencer) or *shUsp8* plasmids for 48 hours. The whole cell lysate was immunoprecipitated with the FK2 antibody and the IPs were analyzed by IB against CHMP1B. Asterisk indicates unspecific band at 35 kDa (see S3C Fig). Double Asterisk corresponds to light and heavy chains of IgG (at 25kDa and 55 kDa, respectively).

The analysis of the whole cell lysate from HEK293T or HeLa cells with an anti-CHMP1B antibody revealed three major bands migrating at 25–28 kDa, 55 kDa and 200 kDa (Fig 3A and S3A Fig). These bands were strongly diminished upon silencing of *CHMP1B* using two independent shRNAs (S3A Fig). These results indicate that endogenous CHMP1B is present as distinct species corresponding to monomers and SDS-PAGE-resistant putative dimers and polymers. Longer heat denaturation of the samples resulted in a loss of the 200 kDa band and an increase of the 55 kDa band, supporting the hypothesis that the high *mw* species corresponds to a SDS-PAGE resistant polymeric form of CHMP1B (S3B Fig). In contrast to the endogenous protein, the recombinant GFP-tagged CHMP1B was only detected as monomeric form possibly due to the presence of the N-terminal GFP tag that may perturb CHMP1B polymerization (see above, Fig 2A).

Then, HEK293T cell lysates were separated on a sucrose gradient as a first step to enrich samples in CHMP1B polymers. Western blot analysis confirmed the presence of polymeric SDS-PAGE resistant CHMP1B in the 20 and 30% sucrose fractions while the putative dimers were present in the upper fractions (0% and 10%) and monomers were not detected by this method (Fig 3B). We then analyzed the 30% sucrose fraction by size exclusion chromatography followed by western blot analysis. This revealed the presence of CHMP1B in fractions 6 to 9 (elution volume 9.5 to 11 ml) (Fig 3C). Because two marker proteins of 669 kDa (thyroglobubin) and 158 kDa (γ-globulin) eluted at 9.8 and 13.2 ml, respectively, we conclude that CHMP1B is part of a ~500 kDa complex. Probing the same fractions with the anti-IST1 antibody showed further that endogenous IST1 is also present in this complex (Fig 3C).

Taken together, our results indicate that endogenous CHMP1B exists as SDS-PAGE resistant polymers that are part of a larger complex that also contains IST1.

### Polymeric CHMP1B is free of ubiquitin

We next analyzed the ubiquitination profile of endogenous CHMP1B. Ubiquitinated proteins from HEK293T cells were immunoprecipitated using the anti-ubiquitin FK2 antibody and further analyzed by western blot using the anti-CHMP1B antibody. As shown above, the same three forms of CHMP1B could be detected in the whole cell lysate (Fig 3D, WCL). In the pool of ubiquitinated proteins, CHMP1B immunoblotting revealed the presence of a major ubiquitinated form of CHMP1B migrating at ~70 kDa that may correspond to an ubiquitinated dimer of CHMP1B (Fig 3D, IP FK2) while the minor band detected at 35 kDa might be non-specific (see S3C Fig). In contrast, the polymeric form of CHMP1B was not found in the ubiquitinated fraction (Fig 3D, IP FK2). Thus, polymers of CHMP1B that are part of the ESCRT-III complex with IST1 are most likely devoid of ubiquitin.

In parallel, endogenous CHMP1B was immunoprecipitated from the HEK293T cell extract and detected by immunoblotting with anti-CHMP1B. Likewise, the monomers, the putative dimers and the polymers could be detected in the whole cell lysate (Fig 3D, WCL). Interestingly, only the polymers and the ubiquitinated, but not the non-ubiquitinated, forms of CHMP1B, were found in the CHMP1B fraction immunoprecipitated from native cell extracts (Fig 3D, IP CHMP1B). Since we used a polyclonal human anti-CHMP1B antibody raised against a peptide covering residues 35 to 84 (Fig 3E), the epitope(s) recognized by this antibody might be completely or partially hidden by the auto-inhibitory helix 6 in the monomeric closed conformation [22] thereby preventing its recognition and immunoprecipitation by the antibody from native cell lysates. We thus suggest that these epitope(s) are exposed for antibody recognition in polymeric as well as in ubiquitinated forms of CHMP1B while they are masked in non-ubiquitinated ones. Taken together, our results show the existence of endogenous ubiquitinated forms of CHMP1B, which may correspond to ubiquitinated dimers, and that CHMP1B polymeric forms are not ubiquitinated.

Finally, we observed that the ubiquitination level of endogenous CHMP1B was higher in partially *Usp8*-silenced cells compared to control cells, strengthening the hypothesis that USP8 deubiquitinates CHMP1B (Fig 3F).

### Dynamic ubiquitination of CHMP1B upon EGF treatment

**Fig 4:**
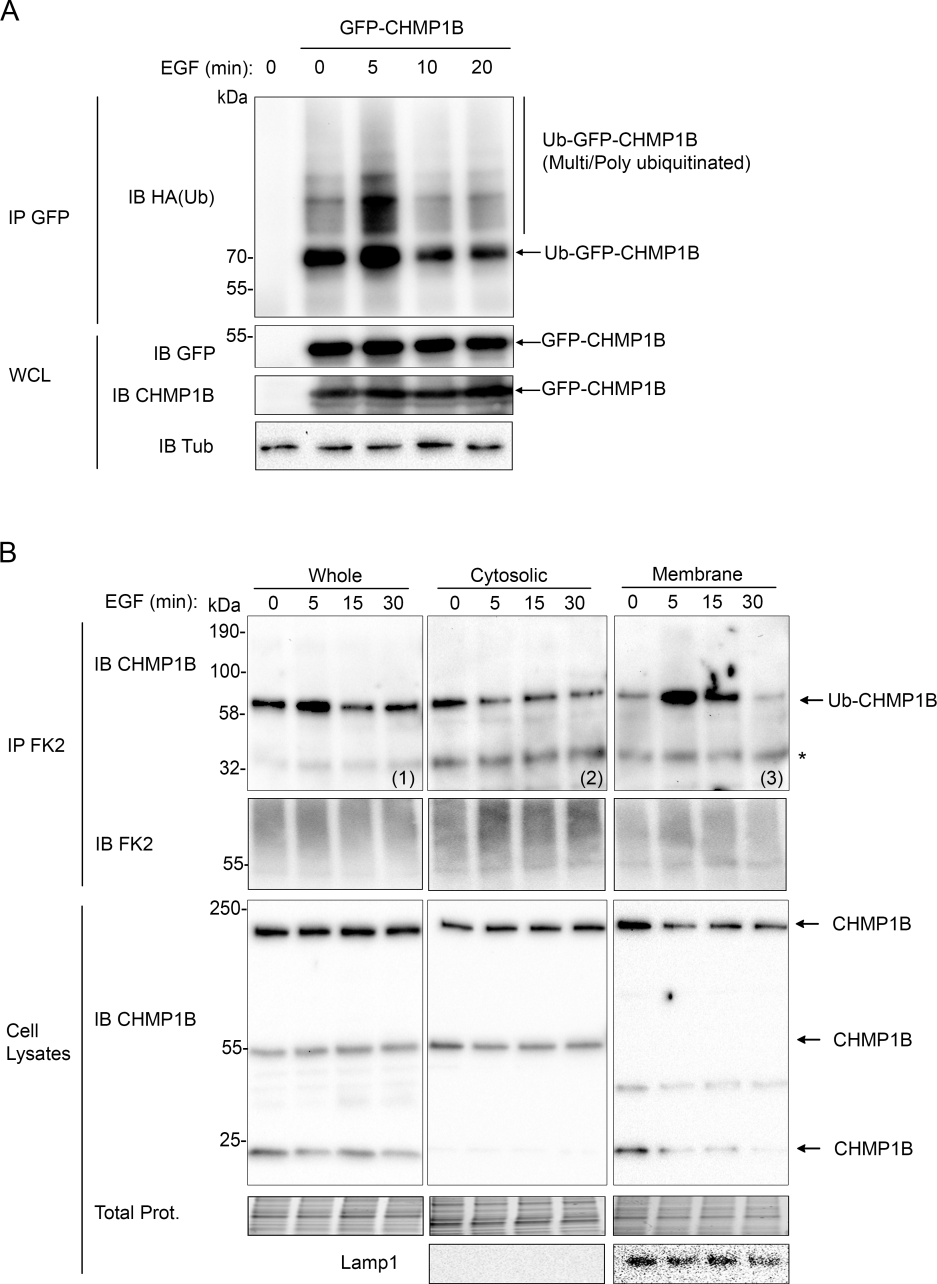
Dynamic ubiquitination of CHMP1B in response to EGF stimulation. **A**: GFP-CHMP1B constructs were transfected into HEK293T cells jointly with HA-ubiquitin (HA-Ub). Cells were starved for 16 hours prior to stimulation by EGF at 100 ng/ml. Immunoprecipitations (IP) were carried out with anti-GFP antibodies of the lysate and proteins were analyzed by immunoblot (IB) using either anti-HA (Ub) or anti-GFP antibodies. Whole cell lysates (WCL) were immunoblotted with indicated antibodies. **B**: Serum starved HEK293T cells were subjected to EGF at 100ng/ml stimulation. Whole cell lysates (Whole) and cell fractions (Cytosolic and Membrane) were prepared as indicated in supplemental methods. Briefly, cell debris and nuclei were eliminated though a brief centrifugation and cell lysates were then re-centrifuged at 13000 x g for 20 min at 4°C. The supernatant was collected as cytosolic fraction while the pellet was re-suspended, briefly centrifuged to eliminate insoluble proteins, and collected as membrane fraction. Ubiquitinated proteins were immunoprecipitated with FK2 antibody from either the WCL (1), the cytosolic (2) or the membrane (3) fractions at the indicated time of EGF stimulation. IPs were analyzed by SDS-PAGE and revealed by IB using anti-CHMP1B or FK2 antibodies. Total proteins are shown. Lamp1 expression was analyzed from supernatant or pellet before IP.

Given the role of the ESCRT machinery in the downregulation of a vast array of receptors in response to their ligands, including EGFR and the pro-inflammatory IL1R (Interleukine 1 Receptor) [64,65], we tested the effect of EGF and IL1β on CHMP1B ubiquitination when added at doses known to induce the internalization of their respective receptors [64,65]. In cells co-expressing GFP-CHMP1B and HA-Ub, a transient increase of the amount of ubiquitinated GFP-CHMP1B was observed at 5 min of stimulation with EGF (Fig 4A). This timing coincides with the onset of the interaction between EGFR and the ESCRT proteins [45]. In the case of IL1β treated cells, a transient accumulation of ubiquitinated GFP-CHMP1B was observed at 10 min of stimulation (S4A Fig). These experiments show that ubiquitination of CHMP1B is dynamically regulated in response to EGF or cytokine stimulation.

We then tested the effect of EGF stimulation on the ubiquitination profile of endogenous CHMP1B. HEK293T cells were treated with 100 ng/ml of EGF and ubiquitinated proteins were immunoprecipitated using the FK2 antibody at different time points. A transient, although modest, increase of the amount of endogenous Ub-CHMP1B putative dimers was observed at 5 min post-EGF stimulation in the FK2 immunoprecipitate from whole cell lysates (Fig 4B, blot (1)). Fractioning of cytosolic (Fig 4B, blot (2)) versus membrane (Fig 4B, blot (3)) fractions prior to immunoprecipitation revealed that the pool of ubiquitinated CHMP1B in response to EGF was strongly enriched in the membrane fraction from 5 to 15 min of EGF stimulation (Fig 4B, blot (3)). Analysis of the corresponding cell fractions prior to immunoprecipitation revealed no significant change in the CHMP1B profile in the whole cell lysates or the cytosolic fraction. In the membrane fraction however, the 55kDa band was not detected as opposed to a new lower band whose molecular nature remains to be determined (Fig 4B). Nuclear versus cytoplasmic fractions were analyzed in the same way and showed that the accumulation of ubiquitinated CHMP1B following EGF stimulation did not correspond to nuclear CHMP1B (S4B Fig).

From these results, we conclude that endogenous CHMP1B is rapidly ubiquitinated upon EGF stimulation resulting in the accumulation of ubiquitinated CHMP1B at cellular membranes.

### Ubiquitination of CHMP1B is required for EGFR degradation

**Fig 5:**
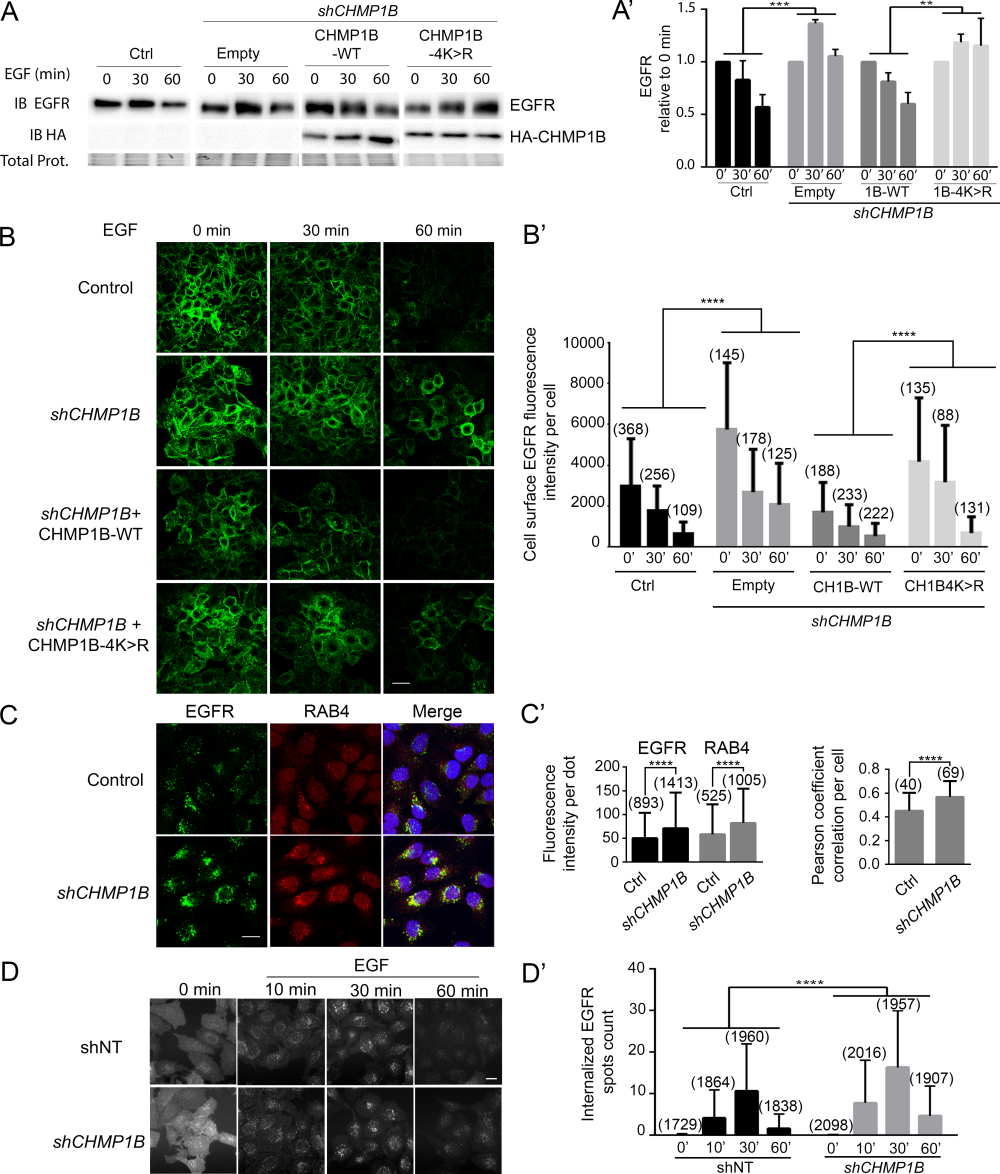
Ubiquitination of CHMP1B is required for EGFR degradation. **A:** Analysis of lysates from HeLa cells expressing or not a shRNA-*CHMP1B* against the 3’UTR region (sh94) and transfected with HA-empty, HA-CHMP1B-WT or HA-CHMP1B-4K>R as indicated. Cells were subjected to 16 hours serum starvation prior to stimulation by EGF and lysed at the indicated time points prior to analysis. Proteins were separated by SDS-PAGE and revealed by immunoblots (IB) using either anti-EGFR or anti-HA antibodies. Total protein amounts are shown. **A’:** Quantification of the amount of EGFR was carried out by densitometry and normalized signals are expressed as a fold-change over the basal EGFR level at time 0 minutes (in the absence of EGF) to circumvent variations in total EGFR amount prior to stimulation. Histograms represent the mean of three independent experiments. Error bars indicate standard deviation. Statistical significance was determined using two-way ANOVA (***p<0.001, **p<0.001). **B:** Confocal images of control or *CHMP1B*-silenced (*shCHMP1B-94*) HeLa cells transfected with HA-empty, HA-CHMP1B or HA-CHMP1B-4K>R. Cells were subjected to serum starvation prior to stimulation by EGF and fixed at the indicated time points. Immunofluorescence staining of non-permeabilized cells was performed to stain specifically plasma membrane EGFR. Scale bar: 40μm. **B’:** Quantification of the intensity of fluorescence per cell was performed using Cell Profiler Software. Vertical axis indicates fluorescence intensity units per cell. Histograms represent the mean intensity measured from individual cells. Error bars indicate standard deviation. The numbers between parentheses indicate the number of cells measured for each experimental condition. Statistical significance was determined using two-way ANOVA (****p<0.0001). Note that CHMP1B-WT expression in *shCHMP1B* context induced an apparent complete rescue of EGFR disappearance from the cell membrane while the immunostaining revealed only about 50% of CHMP1B-WT expressing cells (as shown in S5B Fig). One possible explanation is that a number of transfected cells express low levels of the rescuing CHMP1B protein that are not detectable by immunofluorescence but sufficient for the rescue of *CHMP1B* knock-down. In addition, overexpression of CHMP1B reduced the level of EGFR at the membrane compared to control cells as clearly observed at the starting point (see time 0) which may also account for the apparent total rescue of the phenotype. **C:** Confocal images of HeLa cells or *CHMP1B*-silenced HeLa cells (*shCHMP1B-94*). Cells were subjected to 16 hours serum starvation prior to stimulation by EGF and fixed at 30 min of stimulation. Immunofluorescence staining was performed on permeabilized cells using anti-EGFR and anti-RAB4 antibodies. Scale bar: 20μm. **C’**: Left histogram: EGFR and RAB4 dots were segmented and their intensity measured using the Cell Profiler software. The mean intensity and the standard deviation (error bars) calculated from individual dots are shown. The numbers between parentheses indicate the number of dots measured for each experimental condition. Statistical significance was determined using T-test (****p<0.0001). Right histogram: the Pearson coefficient correlation between EGFR and RAB4 dots was calculated for each cell using the Cell Profiler software. The mean Pearson coefficient and the standard deviation (error bars) calculated from individual cells are shown. The numbers between parentheses indicate the number of cells measured for each experimental condition. Statistical significance was determined using T-test (****p<0.0001). **D, D’**: Control (shNT) and *shCHMP1B-94* silenced HeLa cells (*shCHMP1B*) were serum starved for 16 hours. An antibody directed against the extracellular domain of EGFR was added in the culture medium together with EGF and the EGFR/antibody complex was let allowed to internalize for the indicated time of EGF stimulation before fixation. D: Cells stained with EGFR were imaged by automated ArrayScan microscope. D’: Quantification of EGFR spots was performed using the dedicated software (see methods). Scale bar: 40μm. Statistical significance was determined using two-way ANOVA (****p<0.0001).

In order to investigate the physiological relevance of CHMP1B ubiquitination, we analyzed the EGFR degradation kinetic following EGF stimulation in stably *CHMP1B*-silenced (sh*CHMP1B*) HeLa cells that were transiently transfected with shRNA-resistant CHMP1B-WT or CHMP1B-4K>R constructs (Fig 5A; S5A and S5B Fig). *CHMP1B*-silenced cells exhibited delayed EGFR degradation compared to the control cells (Fig 5A and 5A’). Expressing CHMP1B-WT, but not CHMP1B-4K>R, restored the kinetic of EGFR degradation in *CHMP1B*-silenced cells to the control cell situation (Fig 5A and 5A’).

We then analyzed the plasma membrane expression of EGFR in *CHMP1B*-silenced cells expressing or not the shRNA-resistant versions of either wild-type or 4K>R mutant forms of CHMP1B (Fig 5B and 5B’). The amount of EGFR at the plasma membrane before and after EGF stimulation was followed by immunostaining on fixed, but non-permeabilized cells to better preserve the membrane structure and mostly visualize EGFR located at the plasma membrane. We observed a higher level of plasma membrane EGFR expression in *CHMP1B*-silenced cells compared to control cells both before and at each time point of EGF stimulation (Fig 5B and 5B’). Treatment of control HeLa cells with 100 ng/ml EGF induced the expected progressive removal of EGFR from the plasma membrane in the ensuing 30 min and an almost complete loss of staining at 60 min post-stimulation (Fig 5B and 5B’). In contrast, EGFR staining at the plasma membrane was reduced but still present at 60 min in *CHMP1B-*silenced cells (Fig 5B and 5B’). Interestingly, the expression in *CHMP1B*-silenced cells, of CHMP1B-WT, but not of CHMP1B-4K>R, was able to induce EGFR disappearance from the membrane after EGF stimulation, clearly supporting an important role for CHMP1B ubiquitination in EGFR trafficking (Fig 5B and 5B’). Co-staining of EGFR and RAB4, a marker for early recycling endosomes [66], revealed that both the EGFR amount and the RAB4 staining were higher, with an enhanced correlation of the two signals, in *CHMP1B*-silenced versus control cells (Fig 5C and 5C’).

We further investigated if the internalization process *per se* was defective in *CHMP1B*-silenced cells. To this end, we performed a trafficking assay using an EGFR antibody that recognizes the extracellular domain of EGFR [67] which was directly added to the culture medium on living cells. Then, cells were washed, fixed and secondary antibody was added. This procedure allowed to detect plasma membrane EGFR at time 0 and intracellular EGFR associated with the primary antibody internalized from the cell surface at different time points following EGF stimulation (Fig 5D). The quantification of internalized EGFR over time showed a proper or even enhanced level of internalized EGFR in *CHMP1B*-silenced cells versus control cells (Fig 5D and 5D’). Co-staining with endosomal markers to reveal the early (EEA1), late (Lamp1) and early recycling (RAB4) endosomes indicates that EGFR traffics through these different endosomal compartments although with a different kinetic in *shCHMP1B*-silenced cells compared to control cells (S6 Fig).

Taken together, our results show that EGFR is internalized but most likely less efficiently degraded in *CHMP1B*-silenced cells compared to control cells following EGF stimulation. Moreover, CHMP1B ubiquitination is required for proper EGFR degradation.

### Ubiquitination of CHMP1B is required for *Drosophila* wing development

**Fig 6:**
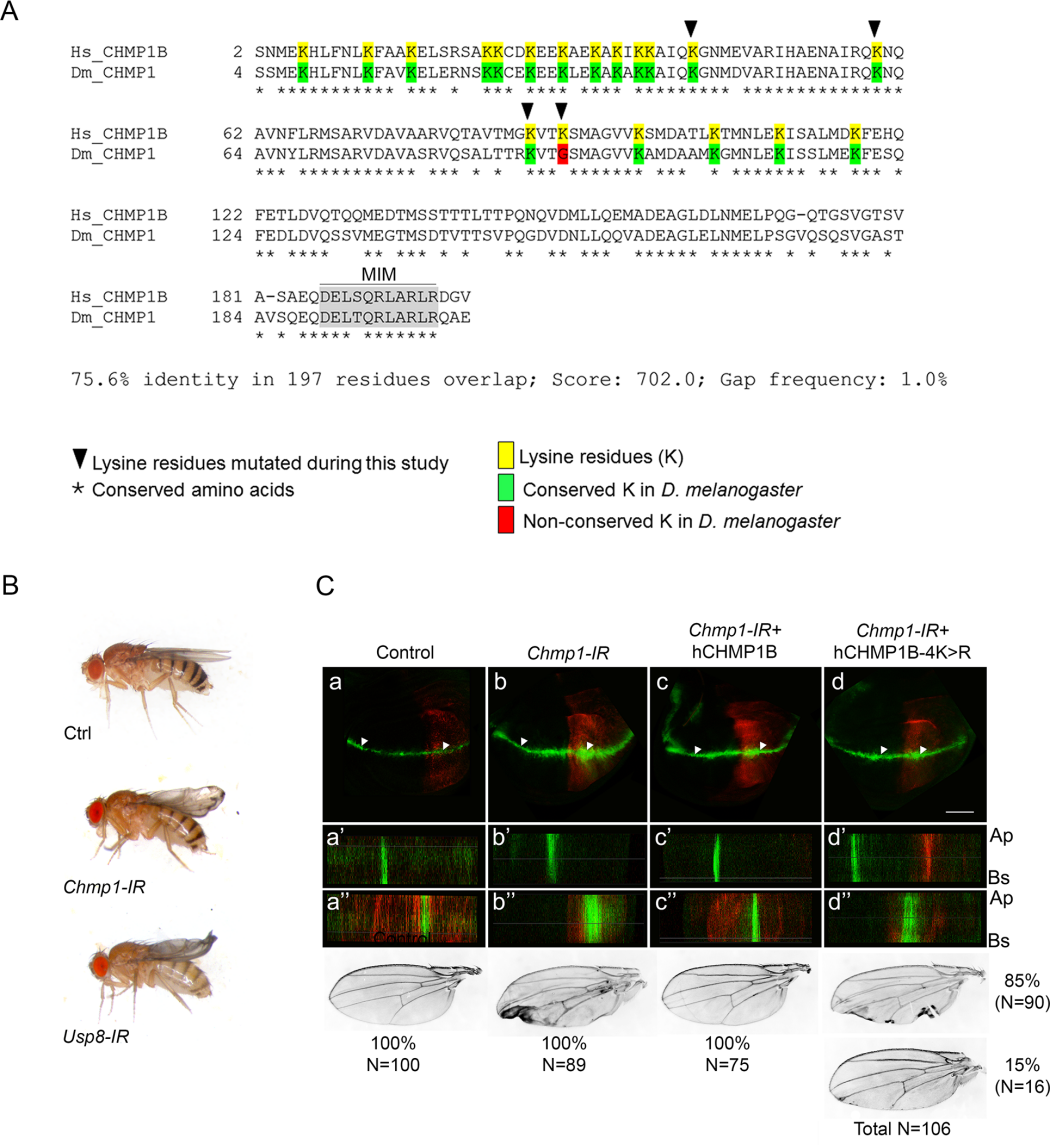
The ubiquitin deficient form of CHMP1B is not functional in *Drosophila*. **A:** Protein sequence alignment of *Drosophila melanogaster* CHMP1 versus its *Homo sapiens* orthologue CHMP1B. Lysine residues in Hs-CHMP1B are marked in yellow. Conserved lysine residues in *Dmel*-CHMP1 are marked in green, the one which is not is marked in red. The four lysine substituted by arginine residues in CHMP1B-4K>R are marked with red arrow-heads. The MIM (MIT interacting domain) is marked in grey. **B:** Pictures of 3 to 5 day-old female flies raised at 25°C expressing the indicated transgenes under the control of the wing MS1096-Gal4 driver. Genotypes are as follows in *w*^*1118*^ background. **Ctrl**: MS1096/+. ***Chmp1-IR***: MS1096/+;UAS-lacZ/+;*UAS-Chmp1-IR/+*. ***Usp8-IR***: MS1096/+;*UAS-Usp8-IR/+;UAS-birA*. The number of flies examined is N>100 in all cases. The phenotypes were 100% penetrant in all cases. **C: Upper panels:** confocal images of dissected late third instar larval wing imaginal discs. EGFP is expressed under the control of Notch Responding Elements (NRE). Myr-RFP is expressed under the control of UAS sequences. Gal4 is expressed under the control of *engrailed* (*en*) promoter. *a*,*b*,*c*,*d*: frontal average Z stack of confocal series. a’ and a” show lateral views of the Z stack indicated by white arrowheads in (a), at the level of either the non-Gal4 expressing area (a’, corresponding to anterior part of the wing imaginal disc) or the Gal4 expressing area (a”, corresponding to the posterior part of the wing imaginal disc). Same configuration for b’, b”, c’, c”, d’, d”. Ap: apical side. Bs: basal side. Magnification objective 63x. Scale bar: 75μm. Bottom panels: pictures of corresponding adult wings. The penetrance of the phenotype is indicated in percentage calculated on (N) flies examined as indicated. Genotypes are as follows: **Control**: *w*^*1118*^*; en-Gal4*, *UAS-Myr-RFP*, *NRE-EGFP/+*. ***Chmp1-IR***: *w*^*1118*^; *en-Gal4*, *UAS-Myr-RFP*, *NRE-EGFP/+*; *UAS-Chmp1-IR/+*. ***Chmp1-IR* + hCHMP1B**: *w*^*1118*^; *en-Gal4*, *UAS-Myr-RFP*, *NRE-EGFP*/*UAS-*hCHMP1B; *UAS-Chmp1-IR/+*. ***Chmp1-IR* + hCHMP1B-4K>R**: *w*^*1118*^; *en-Gal4*, *UAS-Myr-RFP*, *NRE-EGFP*/*UAS-*hCHMP1B-4K>R; *UAS-Chmp1-IR/+*.

To assess the generality of a CHMP1B regulation by the ubiquitin system, we extended our experiments to the *Drosophila melanogaster* model organism. The *Drosophila melanogaster* genome encodes one orthologue of *Hs*USP8, the protein *Dmel*USP8 (syn. *Dme*lUBPY) that shares 45% sequence identity with its human counterpart and a unique CHMP1 protein, *Dmel*CHMP1, displaying 90% identity with human CHMP1B [68]. Three out of the four lysine residues targeted in this work are conserved and the MIM domain is almost identical except for one conservative substitution (Fig 6A).

Ubiquitous silencing of *Chmp1* or *Usp8* in *Drosophila* is lethal before the pupal stage, so we used transgenic fly lines expressing silencing hairpins under the control of the UAS promoter [69] together with the specific wing Gal4 driver MS1096-Gal4 inducing the expression of the UAS constructs in the dorsal wing layer. Consistent with previous studies showing the role of these two genes in wing development [63,68,70], silencing either *Chmp1* or *Usp8* resulted in a similar curved and growth defective wing phenotype (Fig 6B).

During wing development, a complex set of signaling events establishes a row of active Notch pathway cells in the wing larval imaginal disc, at the level of the future wing margin [71,72]. Using an EGFP reporter gene expressed under Notch Responsive Elements (NRE) and the driver *engrailed*-Gal4 (en-Gal4) to induce the silencing construct in the posterior part of the wing imaginal disc, we observed that silencing endogenous *Chmp1* resulted in an enlarged domain of EGFP expression both in the domain of *engrailed* (*en*) expression and slightly beyond (Fig 6C). Defects outside the domain of *shCHMP1* expression is in accordance with a non-cell autonomous role of ESCRT proteins in the regulation of developmental signals [70,73–80]. This resulted in observable wing margin defects at the adult stage (Fig 6C).

Transgenic flies conditionally expressing human transgenes encoding either wild-type *Hs*CHMP1B or *Hs*CHMP1B-4K>R were then generated to test their ability to rescue the wing phenotype induced by *DmelChmp1* silencing. Remarkably, restriction of the Notch signaling domain in larval imaginal discs was restored by expressing wild-type *Hs*CHMP1B resulting in normal adult wings. In contrast, expressing *Hs*CHMP1B-4K>R mutant only partially restored the restriction of Notch activity in the wing margin, resulting in a high proportion of adults presenting wing margin defects (85%) and a few adults with almost normal wings (15%) (Fig 6C). Incomplete rescue by the ubiquitination defective *Hs*CHMP1B-4K>R mutant indicates that modification of CHMP1B by ubiquitin linkage contributes to the protein function in *Drosophila* thus providing evidence for the evolutionary conservation of a ubiquitination-based regulation of *Hs*CHMP1B and *Dmel*CHMP1 proteins.

## Discussion

Presently, little is known about the regulation and activation of human ESCRT-III proteins *in vivo*. Here we show that the ESCRT-III member CHMP1B is regulated by ubiquitin linkage which is tightly controlled by the ubiquitin specific protease USP8. We propose that CHMP1B ubiquitination serves as a checkpoint for spatial and temporal control of its polymerization at endosomes.

We identified the lysine residues K87 and/or K90 situated in the linker region connecting α-helices 2 and 3 of CHMP1B as major site(s) of ubiquitination. Compared to the human protein, K87 is conserved but not K90 in *Drosophila melanogaster* and inversely in *Arabidopsis thaliana* (S7 Fig), pointing out the importance of the presence of at least one lysine residue in this region. This linker region is crucial for the transition from the closed inactive state to the open active polymer conformation of ESCRT-III CHMP1B [22,23]. Notably, in the conversion from the closed to the open conformation, the flexible linker region extends helix 2 of the hairpin (S2A–S2H Fig). We hypothesize that ubiquitination of K87 or K90 residues could possibly induce or stabilize an open conformation of CHMP1B monomers or dimers. In agreement with this hypothesis, we have shown that non-ubiquitinated monomers or dimers of CHMP1B are not recognized by the CHMP1B antibody that targets an epitope presumably masked by the auto-inhibitory helix while ubiquitinated forms of CHMP1B expose the corresponding epitope.

Remarkably, ubiquitinated forms of CHMP1B were not detected as polymers indicating that ubiquitin moieties on CHMP1B may prevent its assembly into ESCRT-III filaments/complexes. We further purified the CHMP1B containing complexes and showed that they are part of a bigger complex containing IST1, an ESCRT-III member previously shown to co-polymerize with CHMP1B *in vitro* [22]. The finding that CHMP1B is free of ubiquitin in these complexes is in accordance with the fact that CHMP1B:IST1 polymerization was observed *in vitro* with non-ubiquitinated recombinant proteins [22]. Furthermore, we have demonstrated that USP8 deubiquitinates CHMP1B. Thus, deubiquitination of CHMP1B by USP8 at the endosomal membrane may favor CHMP1B oligomerization and co-assembly with IST1 *in vivo*.

It is well described that EGF induces the active sorting of the EGFR at the endosomal membranes where it can be either directed to the lysosomal degradation pathway or recycled back to the plasma membrane [81]. Here, we observed that ubiquitinated CHMP1B strongly accumulate on membranes upon EGF stimulation. Therefore, our observation provides strong evidence for a correlation of the transient accumulation of ubiquitinated CHMP1B at the membrane with the activation of EGFR trafficking. Thus, ubiquitination of one or two of the exposed lysine residues may also favor CHMP1B binding to membrane-associated complexes prior to its incorporation in ESCRT-III complexes.

We show that *CHMP1B-*silenced cells present delayed degradation kinetic of EGFR following EGF stimulation. By staining specifically plasma membrane EGFR in non-permeabilized cells, we were also able to observe that *CHMP1B-*silenced cells present a delay in the disappearance of EGFR from the plasma membrane following EGF stimulation. However, the *CHMP1B*-silenced cells present normal or even enhanced internalization of EGFR following EGF stimulation. These results together with the data from the literature showing the role of CHMP1B in MVB biogenesis [82] are consistent with a defect in EGFR sorting, in which decreased lysosomal degradation could be coupled with an enhanced recycling rate of EGFR at the plasma membrane. This hypothesis was further supported by enhanced correlation of EGFR staining with RAB4 following EGF stimulation. While further investigation is needed to understand the exact function of CHMP1B in EGFR sorting, we observed that expression of the CHMP1B-4K>R mutant failed to rescue the degradation kinetic of EGFR while wild-type CHMP1B did rescue it. Although we cannot completely exclude that the K>R mutations impair CHMP1B function by themselves, this substitution is conservative and the side chains of the four lysine residues are solvent exposed in the closed conformation model and in the CHMP1B polymer structure. We thus propose that defective ubiquitination alters CHMP1B function in receptor sorting. Hence, the ubiquitination of CHMP1B would play a major role in the regulation of the sorting of the EGFR at the MVB.

Several functions have been described for CHMP1 proteins. The yeast Did2 protein (CHMP1) was suggested to act in concert with IST1 in the control of Vps4, which disassembles ESCRT-III polymers at a late stage in MVB sorting [83–85]. Furthermore, CHMP1B in complex with IST1 has been implicated in the formation of recycling tubules from endosomes by stabilizing positively curved membrane tubules in overexpressing conditions [22]. These various functions together with the observed dynamic ubiquitination of CHMP1B upon EGF or IL1β treatment could indicate that CHMP1B ubiquitination is part of an ubiquitin-dependent sensing mechanism that might control the fate of receptors towards either recycling or degradation.

The crucial role of both CHMP1B ubiquitination and interaction with USP8 was further confirmed *in vivo* by analyzing its role in *Drosophila melanogaster* wing development. Proper wing development depends on signaling molecules, such as Hedgehog (HH), Wingless (Wg) or the EGFR ligand Vein (Vn), which activate signal transduction pathways through endocytosis of their receptors [41,70,79,86,87]. In this process, CHMP1B-dependent secretion of morphogens is essential [70]. Our results indicate that defective ubiquitination of CHMP1B impairs its normal function in the regulation of the developmental signals controlling Notch activation at the wing margin.

In summary, we propose that dynamic CHMP1B ubiquitination in response to plasma membrane receptor activation and internalization regulates its association to membrane bound complexes and that subsequent ubiquitin hydrolysis allows its incorporation into ESCRT-III polymers exerting their function in receptor sorting at the endosomes. Our results are in line with regulation of protein polymerization by ubiquitin linkage [88]. Furthermore, the finding that CHMP1B is a target of USP8 may shed new light in the future on understanding its contribution to membrane receptor trafficking, resistance to chemotherapy or EGFR stabilization in Cushing’s disease.

## Methods

### Cell lines, cell culture and cell imaging

HEK293T and HeLa cells were purchased from ATCC (LGC Standards, United Kingdom). HEK293T and HeLa cells were cultured in Dulbecco's modified Eagle's medium (DMEM) and RPMI 1640 respectively (Life Technologies) supplemented with 10% heat inactivated fetal bovine serum and 1% Penicillin/Streptomycin mix, and grown in 5% CO2 at 37°C in a humidifier incubator.

Cell transfection, immunostaining and imaging (confocal and high content analysis by automated microscopy) are described in supporting information (S1 Detailed procedures).

### Fly stocks

Flies were raised and crossed at 18 or 25°C using standard procedures. Stocks used for gene silencing are: BL#28906 (*Chmp1*-IR) and VDRC#v107623 (*Usp8*-IR) and neutralizing UAS transgenes on second and third chromosomes are VDRC#3955 (UAS-LacZ) and VDRC#58760 (UAS-BirA), respectively. The drivers used are MS1096 (BL#8860) and enGal4 combined with the Notch signaling reporter gene (BL#30729).

Human CHMP1B wild-type and mutated constructs were sub-cloned into pUAST-attb plasmid using EcoR1 and XhoI as restriction sites. Stable transgenic lines were generated by injection into the fly stock attP40 in order to integrate each rescuing construct at the same genomic location ensuring similar expression levels (genotype y1 x67 c23; PattP40). Therefore, variability in the amounts of recombinant protein are the results of differences in protein stability rather than to variability of transcription levels.

Flies crosses and manipulation are described in supporting information (S1 Detailed procedures).

### DNA manipulation and plasmid construction

The construct expressing HA-Ubiquitin (HA-Ub) was obtained from Dr. Mathias Treier [59]. Mammalian expression constructs of human USP8 were cloned by PCR into pmyc-VN155 (I152L) vector at KpnI/SalI, and human CHMP1B wild-type and mutated sequences were cloned into pHA-VC155 vector at KpnI/SalI. All PCR primers were purchased from Sigma-Aldrich. Constructs expressing FLAG-USP8, FLAG-USP8^C748A^ and FLAG-USP8^S680A^ were kindly provided by Dr. M. Komada [63]. Full length and truncated GFP-tagged constructs of CHMP1B were kindly provided by Dr. M. Maki [57]. GFP-CHMP1B was used as a template to generate substitution of lysine to arginine (K>R) residues at position K42, K59, K87 or/and K90 by site-directed mutagenesis using the QuikChange II Site-Direct Mutagenesis Kit (Agilent Technologies). GFP-CHMP1B and GFP-CHMP1B truncated constructs were used as a template to generate HA- or VN- and VC- CHMP1B tagged constructs. Flag-USP8 construct was used as a template to amplify the fragment at position 3311: 5’-AATCTTCAGCAGCTTATATCC-3’ which was cloned into the pSilencer plasmid to generate the silencing construct sh*Usp8*. Silencing *CHMP1B* in HeLa cells was achieved by stable transfection with shRNA-CHMP1B TRCN0000159294(sh-94) (targets 3’UTR region) or shRNA-CHMP1B TRCN0000165547(sh-47) (targets CDS region) from Mission Sigma shRNA library. Cells stably transfected with non-target shRNA (indicated as Ctrl or shNT) were used as controls.

### EGFR trafficking assay

RPMI culture medium (time 0) or EGF diluted at 100 ng/ml in RPMI/0.5% BSA was added to serum-starved HeLa cells together with a primary antibody specifically directed against the extracellular part of the EGFR (ATCC mAb Hybridoma 108, 1/100). EGFR bound to the antibody was let allowed to internalize for 10, 30 or 60 min of EGF stimulation. Then cells were fixed, permeabilized and immunostained as described in supporting information (S1 Detailed procedures).

**Cell lysis, fractioning, sucrose gradient, gel filtration, immunoblotting and immunoprecipitation methods** are described in supporting information (S1 Detailed procedures).

## Supporting information

S1 Fig Expression of USP8 and CHMP1B BiFC constructs. HEK293T cells were transfected with empty vectors or with Myc-VN-USP8 and HA-VC-CHMP1B-WT and truncated constructs, as indicated. Whole cell lysates were analyzed by immunoblot (IB) using either anti-Myc or anti-HA antibodies to reveal transfected constructs. Total proteins are shown.
(TIF)

S2 Fig CHMP1B conformation and ubiquitination pattern. **A-H:** Structural model of CHMP1B conformational change. Lysine residues are shown as sticks. Alpha helices are numbered and colored as indicated. (A) CHMP1B closed conformation. (B) Side view of the CHMP1B closed conformation. (C, D) Close-up views showing the positions of K42, K59, K87, K90, possibly implicated in ubiquitination. (E) Structure of CHMP1B open conformation present in the CHMP1B polymer. (F) Side view of CHMP1B open conformation. (G, H) Close-up views of the putatively ubiquitinated lysine residues in the open conformation.
**I:** Wild-type or mutated GFP-CHMP1B constructs were transfected into HEK293T cells jointly with HA-ubiquitin (HA-Ub). Immunoprecipitations (IP) were carried out with anti-GFP antibodies after strong denaturation of the lysate and proteins were analyzed by immunoblot (IB) using either anti-HA (Ub) or anti-GFP antibodies.
**J:** GFP-CHMP1B constructs were transfected into HEK293T cells jointly with HA-ubiquitin (HA-Ub) and the indicated USP8 constructs. Immunoprecipitations (IP) were carried out with anti-GFP antibodies after strong denaturation of the lysate and proteins were analyzed by immunoblot (IB) using either anti-K48 or anti-HA (Ub) or anti-GFP (control) antibodies. Whole cell lysate was analyzed by immunoblot with anti-GFP, anti-K48 or anti-Tubulin (Tub).
**K:** Same experiments were performed in HEK293T in presence or absence of proteasomal activity blocker MG132. IP GFP were analyzed by immunoblot (IB) using either anti-HA (Ub) or anti-GFP antibodies. Whole cell lysates were analyzed by immunoblot with anti-Flag or anti-Tubulin (Tub) antibodies.
(TIF)

S3 Fig CHMP1B expression. **A:** Analysis of lysates from HeLa cells expressing or not a shRNA-CHMP1B against 3’UTR region (sh94) or the CDS (sh47). Proteins were separated by SDS-PAGE and revealed by IB using anti-CHMP1B on cut membranes to improve the efficiency of the antibody and allow the detection of oligomers.
**B:** Non-treated HEK293T cell lysates were incubated at 95°C during the indicated times in the presence of Laemmli buffer. Proteins were separated by SDS-PAGE and revealed by IB using anti-CHMP1B.
**C:** IB controls: HEK293T cell lysates were either subjected to immunoprecipitation with anti-FLAG or FK2 antibodies, or incubated with Protein G-Sepharose beads alone. Proteins were then separated by SDS-PAGE and revealed by IB, using anti-CHMP1B (IP Flag lane, primary antibody control) or without primary antibodies (no IgG lanes, secondary antibody control).
(TIF)

S4 Fig CHMP1B ubiquitination in response to IL1β or EGF. **A:** HEK293T cells transfected with HA-Ub and GFP-CHMP1B were stimulated with 10 ng/ml of IL1β 48 hours after transfection. At indicated times, cells were subjected to strong denaturation lysis and GFP-CHMP1B was immunoprecipitated from the cleared lysates using anti-GFP antibodies. Immunoprecipitated proteins were analyzed by Western blot using anti-GFP or anti-HA (Ub) antibodies. The band indicated with an asterisk (*) corresponds to IgG heavy chains.
**B.** HEK293T were subjected to EGF stimulation and cell lysates were fractionated at different time points in cytoplasmic (Cytosol+Membrane (Mb)) and nuclear (Nucleus) fractions. All ubiquitinated proteins were immunoprecipitated with the FK2 antibody and analyzed by immunoblot using anti-CHMP1B antibodies. Cell fractions were analyzed with Lamp1 and histone H2B antibodies prior to IP as markers of cytoplasmic and nuclear fractions, respectively.
(TIF)

S5 Fig CHMP1B extinction in shRNA lines and expression of silencing resistant constructs. **A:** HeLa cells were stably transduced with shRNA non-target, shRNA-CHMP1B TRCN0000159294 (targets 3’UTR region; indicated shCHMP1B-94) or shRNA-CHMP1B TRCN0000165547 (targets CDS region; indicated shCHMP1B-47) from Mission Sigma shRNA library. Cells were then transfected with GFP-CHMP1B construct for 48 hours and whole lysates were analyzed by immunoblot using anti-CHMP1B. Endogenous oligomers and monomers are indicated. Note that signal corresponding to CHMP1B endogenous putative dimers is masked by the over-expressed GFP-CHMP1B.
**B:** HeLa cells stably transduced with shRNA non-target, shRNA-CHMP1B-94 (targets 3’UTR region) were transfected with HA-CHMP1B-WT and 4K>R constructs for 48 hours, stained with anti-HA antibody and observed by confocal imaging to reveal expression of the rescuing transgenes.
(TIF)

S6 Fig EGFR trafficking through endosomal compartments. Control (shNT) and *shCHMP1B-94* silenced HeLa cells (*shCHMP1B*) were serum starved for 16 hours. An antibody directed against the extracellular domain of EGFR was added in the culture medium together with EGF and the EGFR/antibody complex was let allowed to internalize for the indicated time of EGF stimulation before fixation. Co-staining of endosomal markers was performed using standard procedures. A-C: Merged pictures of cells co-stained with EGFR and the indicated markers. EGFR staining is in red and endosomal markers (respectively EEA1, LAMP1 and RAB4) are in green, DNA is in blue (revealed by Hoechst). A’-C’ Quantifications of the overlapping area between EGFR spots and indicated markers (in μm^2^) were performed using the dedicated software (see methods). Scale bar: 40μm. Statistical significance was determined using two-way ANOVA (****p<0.0001).
(TIF)

S7 Fig Protein sequences comparison. Clustal analysis of the protein sequences of Vps46.1 of *Arabidopsis thaliana*, CHMP1B of *Homo sapiens* and CHMP1 of *Drosophila melanogaster*. Conserved lysine residues at position 87 and 90 (in the human sequence) are underlined in yellow, non-conserved lysine residues are in red.
(TIF)

S1 Detailed procedures (DOCX)
