# Supplementary figures and images for: CHMP1B is a target of USP8/UBPY regulated by ubiquitin during endocytosis
Source: PLoS Genet. 2018 Jun 22;14(6):e1007456. doi: 10.1371/journal.pgen.1007456 (PMC6033466; doi:10.1371/journal.pgen.1007456)

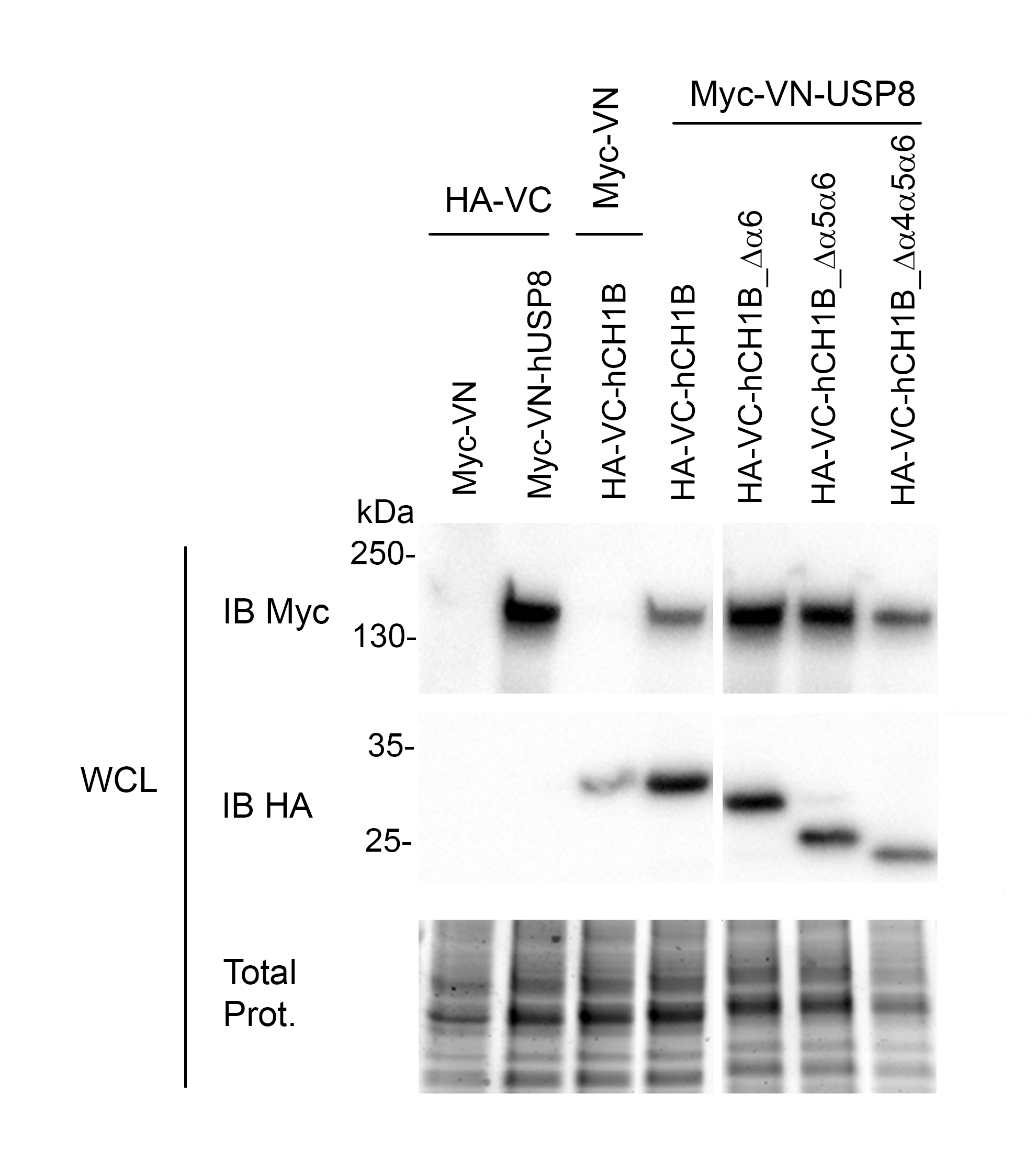

Supplement: S1 Fig — HEK293T cells were transfected with empty vectors or with Myc-VN-USP8 and HA-VC-CHMP1B-WT and truncated constructs, as indicated. Whole cell lysates were analyzed by immunoblot (IB) using either anti-Myc or anti-HA antibodies to reveal transfected constructs. Total proteins are shown. (TIF) [file pgen.1007456.s001.tif]

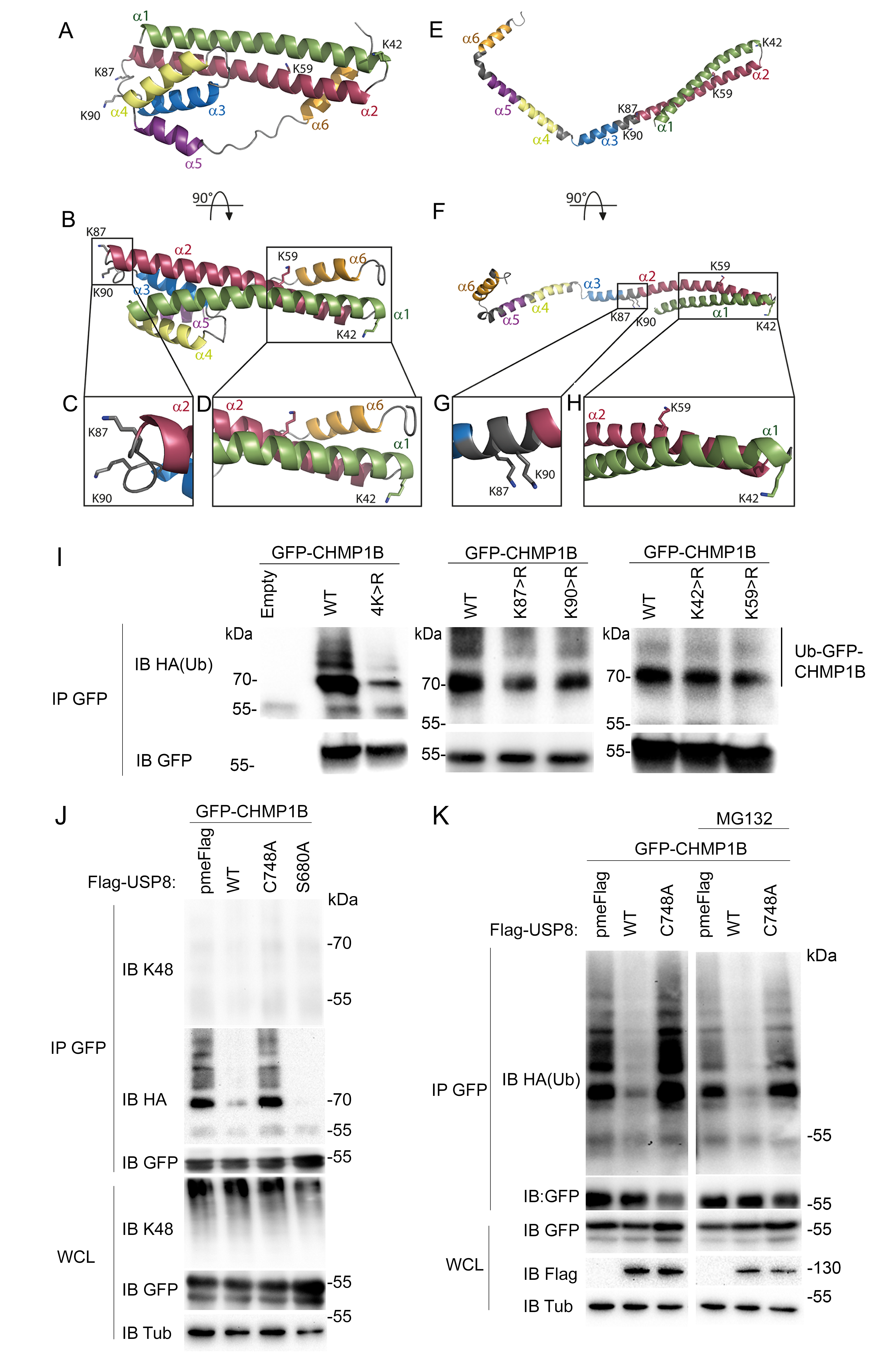

Supplement: S2 Fig — A-H: Structural model of CHMP1B conformational change. Lysine residues are shown as sticks. Alpha helices are numbered and colored as indicated. (A) CHMP1B closed conformation. (B) Side view of the CHMP1B closed conformation. (C, D) Close-up views showing the positions of K42, K59, K87, K90, possibly implicated in ubiquitination. (E) Structure of CHMP1B open conformation present in the CHMP1B polymer. (F) Side view of CHMP1B open conformation. (G, H) Close-up views of the putatively ubiquitinated lysine residues in the open conformation. I: Wild-type or mutated GFP-CHMP1B constructs were transfected into HEK293T cells jointly with HA-ubiquitin (HA-Ub). Immunoprecipitations (IP) were carried out with anti-GFP antibodies after strong denaturation of the lysate and proteins were analyzed by immunoblot (IB) using either anti-HA (Ub) or anti-GFP antibodies. J: GFP-CHMP1B constructs were transfected into HEK293T cells jointly with HA-ubiquitin (HA-Ub) and the indicated USP8 constructs. Immunoprecipitations (IP) were carried out with anti-GFP antibodies after strong denaturation of the lysate and proteins were analyzed by immunoblot (IB) using either anti-K48 or anti-HA (Ub) or anti-GFP (control) antibodies. Whole cell lysate was analyzed by immunoblot with anti-GFP, anti-K48 or anti-Tubulin (Tub). K: Same experiments were performed in HEK293T in presence or absence of proteasomal activity blocker MG132. IP GFP were analyzed by immunoblot (IB) using either anti-HA (Ub) or anti-GFP antibodies. Whole cell lysates were analyzed by immunoblot with anti-Flag or anti-Tubulin (Tub) antibodies. (TIF) [file pgen.1007456.s002.tif]

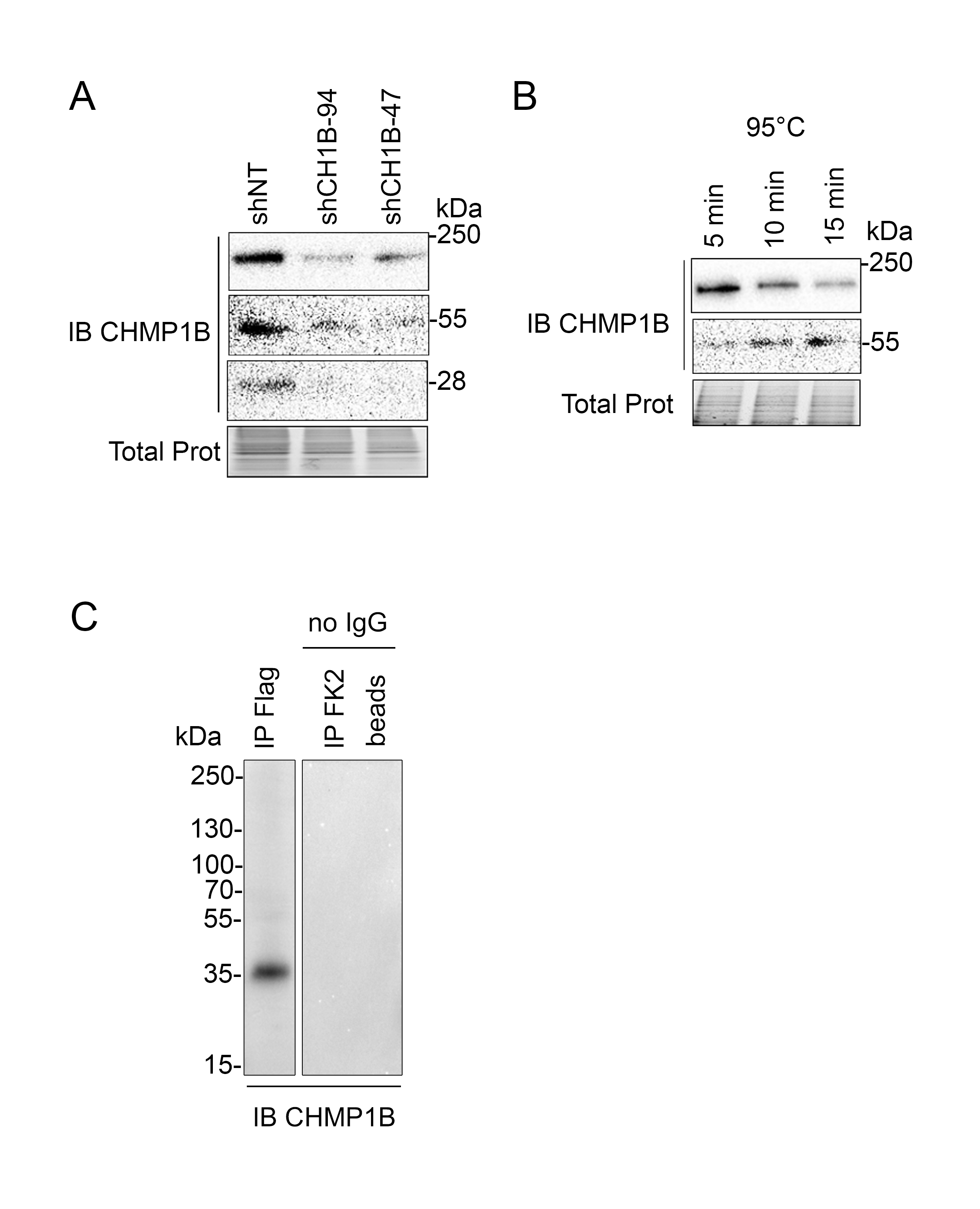

Supplement: S3 Fig — A: Analysis of lysates from HeLa cells expressing or not a shRNA-CHMP1B against 3’UTR region (sh94) or the CDS (sh47). Proteins were separated by SDS-PAGE and revealed by IB using anti-CHMP1B on cut membranes to improve the efficiency of the antibody and allow the detection of oligomers. B: Non-treated HEK293T cell lysates were incubated at 95°C during the indicated times in the presence of Laemmli buffer. Proteins were separated by SDS-PAGE and revealed by IB using anti-CHMP1B. C: IB controls: HEK293T cell lysates were either subjected to immunoprecipitation with anti-FLAG or FK2 antibodies, or incubated with Protein G-Sepharose beads alone. Proteins were then separated by SDS-PAGE and revealed by IB, using anti-CHMP1B (IP Flag lane, primary antibody control) or without primary antibodies (no IgG lanes, secondary antibody control). (TIF) [file pgen.1007456.s003.tif]

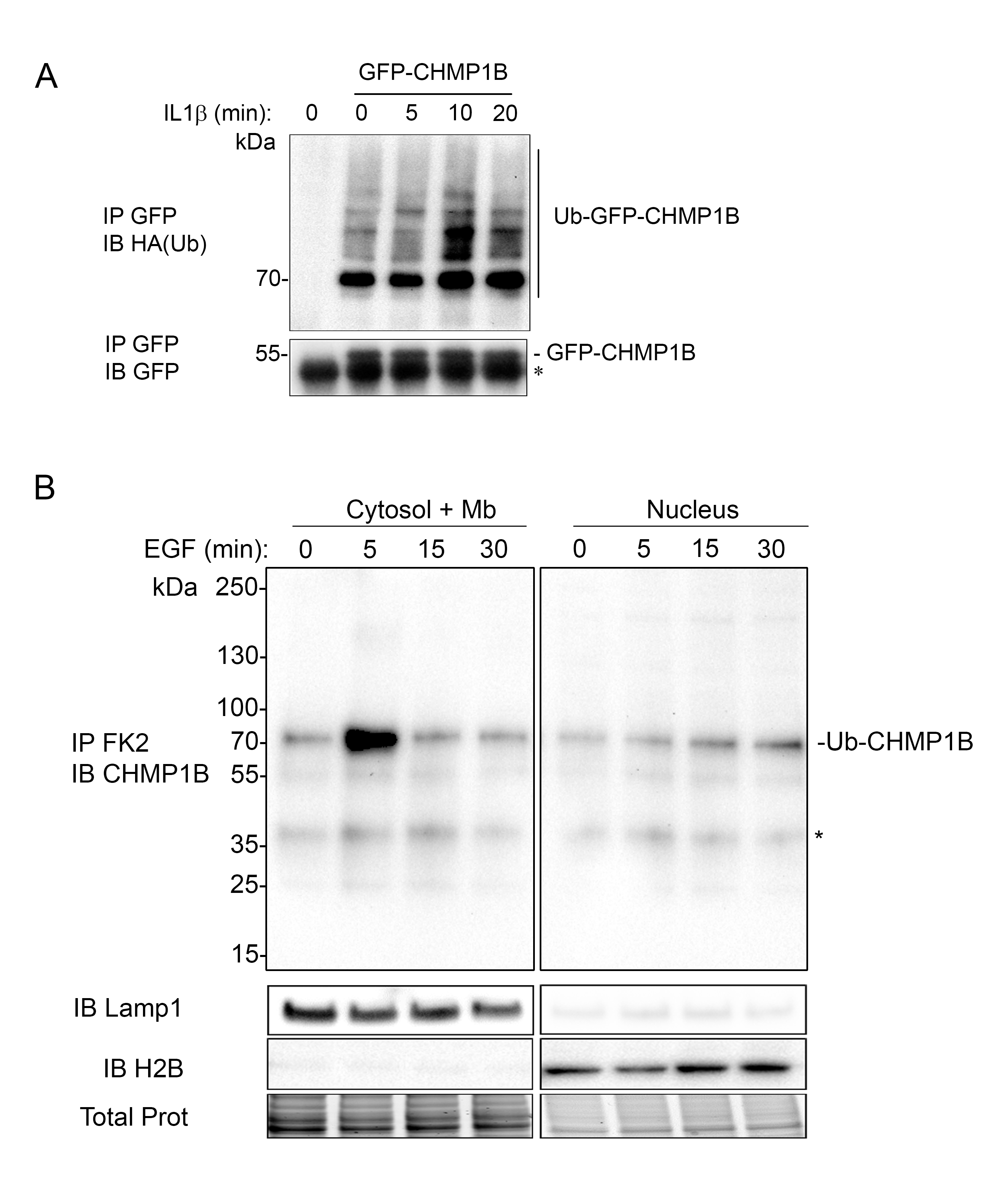

Supplement: S4 Fig — A: HEK293T cells transfected with HA-Ub and GFP-CHMP1B were stimulated with 10 ng/ml of IL1β 48 hours after transfection. At indicated times, cells were subjected to strong denaturation lysis and GFP-CHMP1B was immunoprecipitated from the cleared lysates using anti-GFP antibodies. Immunoprecipitated proteins were analyzed by Western blot using anti-GFP or anti-HA (Ub) antibodies. The band indicated with an asterisk (*) corresponds to IgG heavy chains. B. HEK293T were subjected to EGF stimulation and cell lysates were fractionated at different time points in cytoplasmic (Cytosol+Membrane (Mb)) and nuclear (Nucleus) fractions. All ubiquitinated proteins were immunoprecipitated with the FK2 antibody and analyzed by immunoblot using anti-CHMP1B antibodies. Cell fractions were analyzed with Lamp1 and histone H2B antibodies prior to IP as markers of cytoplasmic and nuclear fractions, respectively. (TIF) [file pgen.1007456.s004.tif]

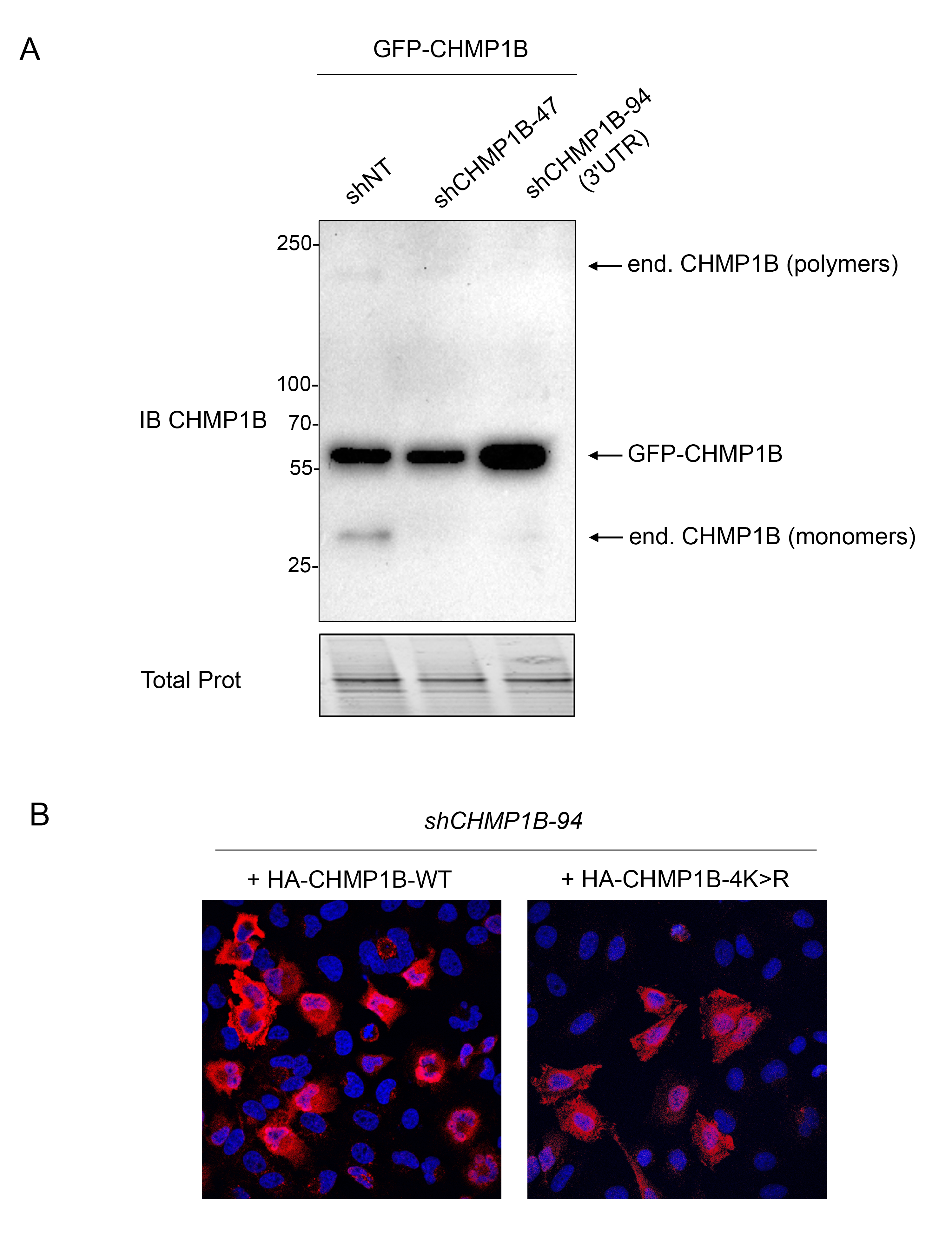

Supplement: S5 Fig — A: HeLa cells were stably transduced with shRNA non-target, shRNA-CHMP1B TRCN0000159294 (targets 3’UTR region; indicated shCHMP1B-94) or shRNA-CHMP1B TRCN0000165547 (targets CDS region; indicated shCHMP1B-47) from Mission Sigma shRNA library. Cells were then transfected with GFP-CHMP1B construct for 48 hours and whole lysates were analyzed by immunoblot using anti-CHMP1B. Endogenous oligomers and monomers are indicated. Note that signal corresponding to CHMP1B endogenous putative dimers is masked by the over-expressed GFP-CHMP1B. B: HeLa cells stably transduced with shRNA non-target, shRNA-CHMP1B-94 (targets 3’UTR region) were transfected with HA-CHMP1B-WT and 4K>R constructs for 48 hours, stained with anti-HA antibody and observed by confocal imaging to reveal expression of the rescuing transgenes. (TIF) [file pgen.1007456.s005.tif]

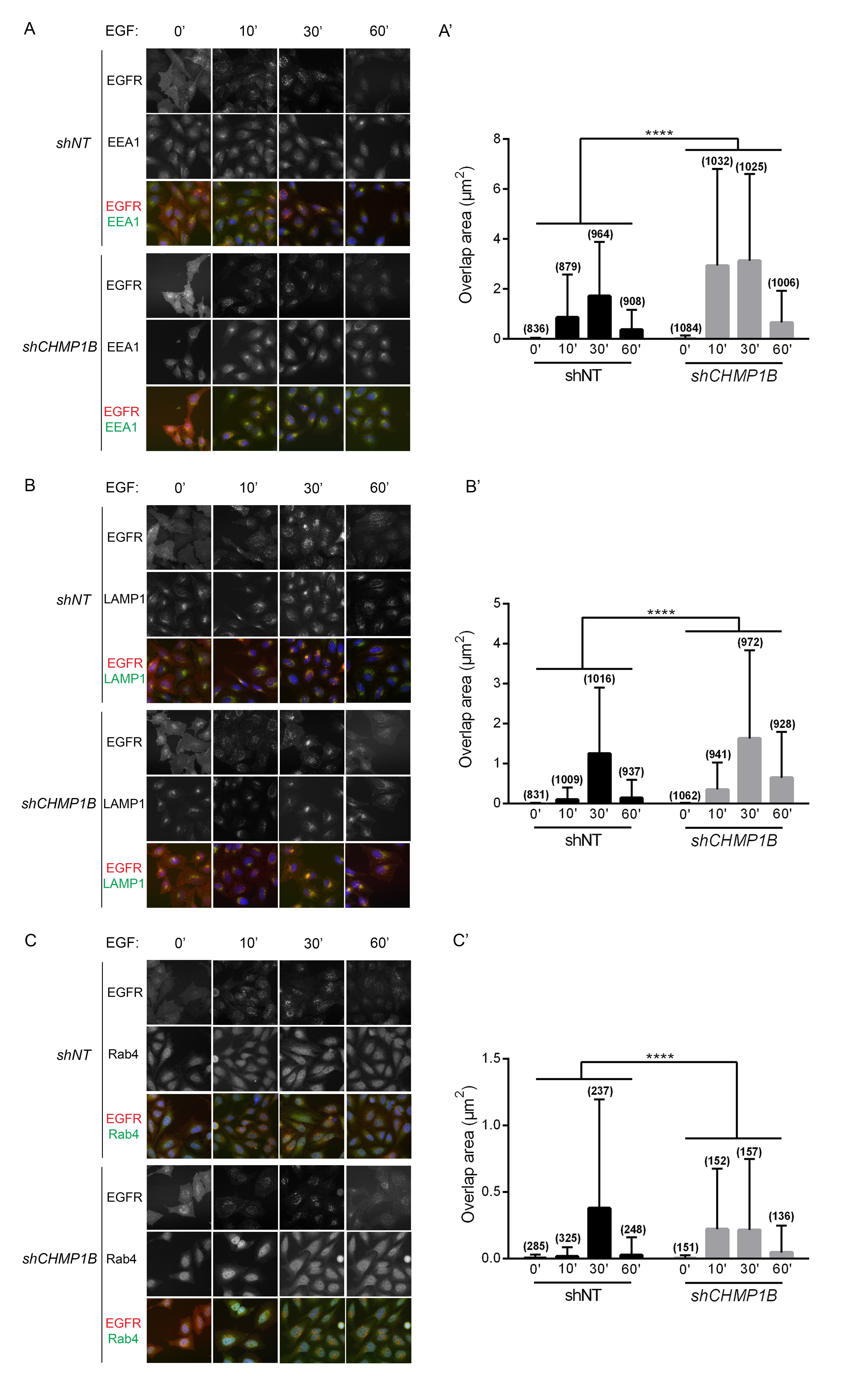

Supplement: S6 Fig — Control (shNT) and shCHMP1B-94 silenced HeLa cells (shCHMP1B) were serum starved for 16 hours. An antibody directed against the extracellular domain of EGFR was added in the culture medium together with EGF and the EGFR/antibody complex was let allowed to internalize for the indicated time of EGF stimulation before fixation. Co-staining of endosomal markers was performed using standard procedures. A-C: Merged pictures of cells co-stained with EGFR and the indicated markers. EGFR staining is in red and endosomal markers (respectively EEA1, LAMP1 and RAB4) are in green, DNA is in blue (revealed by Hoechst). A’-C’ Quantifications of the overlapping area between EGFR spots and indicated markers (in μm2) were performed using the dedicated software (see methods). Scale bar: 40μm. Statistical significance was determined using two-way ANOVA (****p<0.0001). (TIF) [file pgen.1007456.s006.tif]

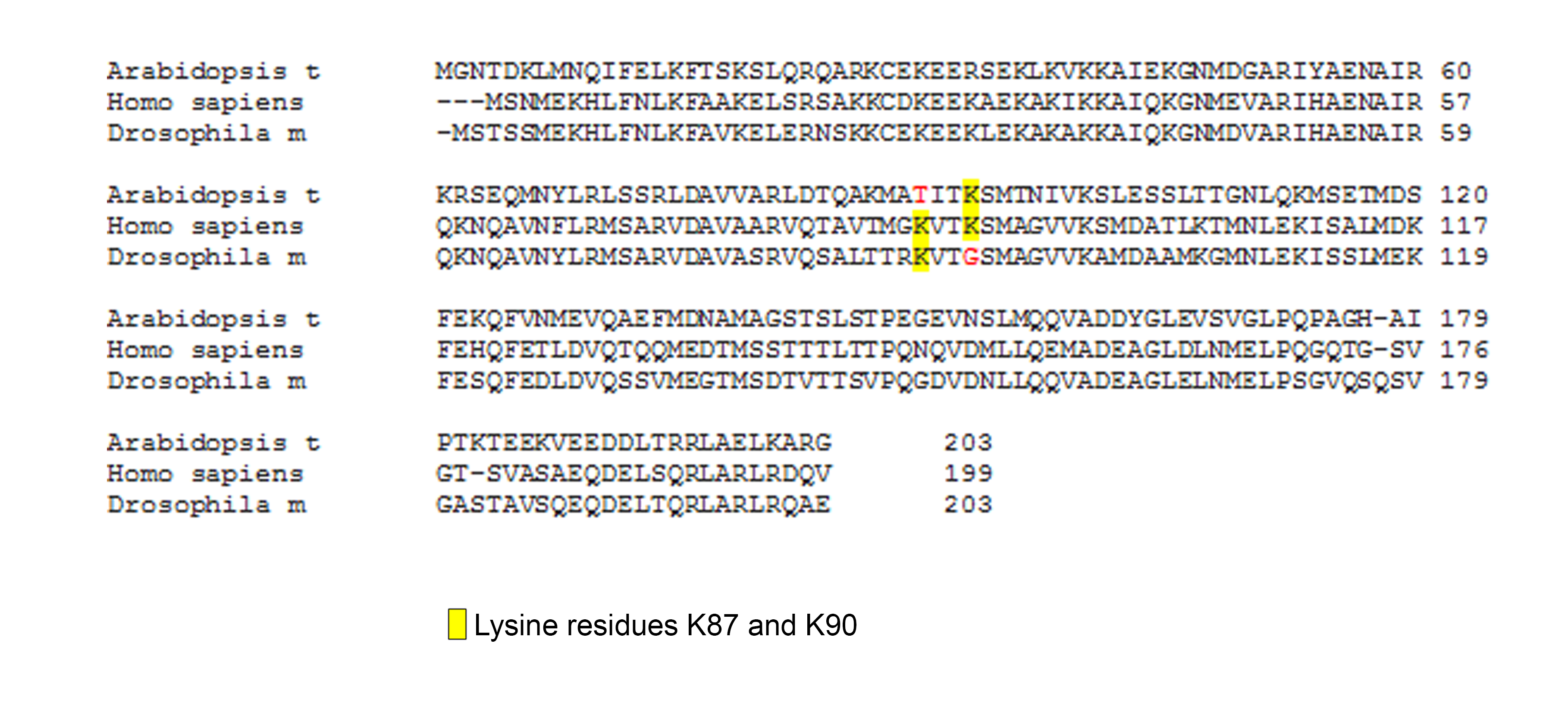

Supplement: S7 Fig — Clustal analysis of the protein sequences of Vps46.1 of Arabidopsis thaliana, CHMP1B of Homo sapiens and CHMP1 of Drosophila melanogaster. Conserved lysine residues at position 87 and 90 (in the human sequence) are underlined in yellow, non-conserved lysine residues are in red. (TIF) [file pgen.1007456.s007.tif]
