# Supplementary material for: CHMP1B is a target of USP8/UBPY regulated by ubiquitin during endocytosis
Source: PLoS Genet. 2018 Jun 22;14(6):e1007456. doi: 10.1371/journal.pgen.1007456 (PMC6033466; doi:10.1371/journal.pgen.1007456)
Supplement: S1 Detailed procedures — (DOCX) [file pgen.1007456.s008.docx]

**Crespo-Yanez C. et al., Supporting Information**

**S1_Detailed Procedures**

**Cell Culture**

**Cell culture and Transfection.** HEK293T and HeLa cells transfections were performed at 50% confluence 24 hours after seeding using FuGENE HD (Promega) according to the manufacturer’s recommendations at ratio 2:1 respectively. The cells were recovered 24-48h after transfection. Serum starvation was performed for 16 hours prior to the stimulation by EGF or IL1β.

**Cell Imaging**

**Bimolecular Fluorescence Complementation (BiFC).** BiFC was used to characterize *in cellulo* the interaction between USP8 and CHMP1B proteins in HeLa cells. Cells were seeded at 8 x 10^3^ per well in 96-well clear bottom tissue culture plates (#655090, Greiner) and allowed to adhere overnight. Cells were co-transfected with 0.16 *µ*g of Myc-VN-USP8 and 0.04 *µ*g of HA-VC-CHMP1B WT or truncated constructs (these amounts of DNA were adjusted to avoid too high levels of recombinant protein expression and the formation of aggregates). Cells were incubated to allow adequate development of Venus fluorescent protein (VFP) for 24h, fixed in PBS/4% paraformaldehyde for 15 min at room temperature (RT), permeabilized 5 min in PBS with 0.5% Triton-X100. After 1 h saturation in PBS/1% BSA, cells were immunostained for 1 h with rat anti-HA and mouse anti-Myc primary antibodies diluted at 1/250 and 1/100 respectively in PBS/BSA 1%. To evaluate VFP co-localization with endosomal compartments, rabbit primary antibodies against EEA-1 or Lamp1 were used at 1/400 dilution. Cells were washed 3 times with PBS/0.1% Tween-20, then incubated for 1 hour with anti-rat Cy3 and anti-mouse Cy5 secondary antibodies, together with Hoechst 33342 for DNA staining, at a 1/1000 dilution, or with goat anti-rabbit Cy3 and Hoechst at 1/1000 for co-localization experiment. Cells were washed 3 times with PBS/ 0.1% Tween-20 and PBS/50% glycerol was added before image acquisition, as described in Quantification section.

**Immunostaining.** HeLa cells were washed in PBS, fixed with 4% PFA during 15 min at RT before being permeabilized in PBS/0.5% Triton-X100 for 5 min at RT. This permeabilization step was omitted in the case of visualization of cell surface EGFR (for confocal imaging (Fig 5 B)). Cells were then washed 3 times in PBS for 10 min at RT, processed for saturation in PBS/5% BSA for one hour at RT and incubated overnight at 4°C with the primary antibodies diluted in PBS/1%BSA (EGFR, 1/400; EEA1, 1/400; Lamp1, 1/400; RAB4, 1/300). Cells were washed 3 times with PBS/0.1% Tween-20 during 10 min at RT with soft agitation, incubated for 1h with the Cy3-, Cy-5 or A488-labeled secondary antibody at 1/1000 in PBS/1%BSA and finally washed 3 times in PBS/0.1% Tween-20 during 10 min at RT. To label the nuclei, cells were incubated with Hoechst 33342 diluted 1/1000 in PBS for 5 min at RT. Cells were maintained in PBS/glycerol (50%) at 4°C before automated imaging or mounted in DaKo fluorescent mounting media for confocal imaging.

**Confocal Imaging.** The samples were imaged with a 63x magnification (oil immersion) using a Leica TCS-SP2 confocal microscope and the LCS software keeping the same acquisition settings for all the conditions.

**Biochemistry**

**Immunoblotting and Immunoprecipitation.** Cell lysis was performed in RIPA buffer (50 mM Tris-HCl, 150 mM NaCl, 1 mM EDTA, 1% IGEPAL, 0.5% sodium deoxycholate) supplemented with a protease inhibitor cocktail (CALBIOCHEM) and, for the ubiquitination assays, with a pan-deubiquitinases inhibitor PR-619 (Sigma-Aldrich). For ubiquitination detection in the presence of transfected HA-Ub, cells were first lysed in conditions of strong denaturation in RIPA supplemented with 1% SDS, boiled at 100°C for 5 min and then diluted in RIPA to obtain a final SDS concentration of 0.1%.

For immunoprecipitation assays, about 300 µg of total cell lysate were used for GFP immunoprecipitation, and 1 mg for CHMP1B or ubiquitin immunoprecipitation. Lysates were first precleared with Protein A or G-Sepharose beads alone (Sigma-Aldrich) for 4 hours at 4°C with rotation. Precleared lysates were then incubated with the indicated antibody (anti-GFP: 3 µg; anti-CHMP1B: 3µg; anti-Ub FK2: 6 µg), and protein A or G-Sepharose beads overnight at 4°C for precipitation of immune complexes. After 4 washes of the beads with RIPA buffer, bound proteins were eluted using Laemmli Sample Buffer (BioRad) supplemented with 10% of β-mercaptoethanol and heated at 95°C for 5 min.

Cell lysates were diluted with Laemmli Sample Buffer 4x (BioRad) supplemented with 10% of β-mercaptoethanol, heated and directly used for immunoblotting.

Western blots were performed as follows: protein lysates and eluates were separated in SDS-PAGE gels (Criterion TGX Stain Free; BioRad). Total proteins were detected directly in the gels using a Chemidoc apparatus (BioRad) and the ImageLab Stain Free Gel protocol. Proteins were transferred to a nitrocellulose or PVDF membrane (BioRad).

**Subcellular Fractioning.** To separate soluble (cytosolic proteins) fraction from insoluble (membrane proteins) fraction, HEK293T cells were harvested, suspended in cold PBS (supplemented with a protease inhibitor cocktail (Calbiochem) and DUB inhibitor PR-619 (Sigma-Aldrich)) and homogenized by passing cells 20 times through a 26-gauge needle. To separate the cytosolic fraction from the membrane one, the cell lysate was first briefly centrifuged at 1000 x g for 5 min at 4°C to eliminate cell debris and nuclei. Then the supernatant was centrifuged at 13000 x g for 20 min at 4°C and the second supernatant was collected as the cytosolic fraction while the pellet was re-suspended using the same volume of PBS with 1% Triton-X100 and incubated in a shaker at 4°C for 10 min. Insoluble proteins were removed by a 5 min centrifugation step at 500 x g at 4°C and the last supernatant was kept as the membrane fraction.

For the nuclear and cytosolic extraction, NE-PER Nuclear and Cytosolic Extraction Reagents Kit (#78835; Thermo scientific, France) was used following the protocol provided by the manufacturer.

**Sucrose Gradient and gel filtration.** HEK293T cells were lysed in RIPA buffer (50 mM Tris-HCl, 150 mM NaCl, 1 mM EDTA, 1% IGEPAL, 0.5% sodium deoxycholate) and the resulting lysate was analyzed by step sucrose gradient (0-10-20-30-40-60 %) centrifugation at 76,000 x *g* for 17h at 4°C. The 30% fraction of the sucrose gradient was concentrated and further separated on a Superdex 200 column in the same RIPA buffer. 0.5 ml fractions were analysed by western blot analysis with the anti-CHMP1B and the anti-IST1 antibodies. Marker proteins thyroglobulin, 670kDa and γ-globulin 158 kDa were separated on the same column.

**Flies’ manipulation**

**Fly crosses.** The MS1096-Gal4 (BL#8696) driver line was used to induce the expression of the Gal4 protein in the dorsal epithelial sheet layer of the wing. Rescue experiments were then carried out by expressing wild type or mutated constructs of human *CHMP1B* together with the *DmelChmp1*-IR transgene. Rescue experiments were carried out balancing the amount of UAS driving sequences by using VDRC#3955 (UAS-LacZ) and VDRC#58760 (UAS-BirA) neutral UAS sequences. Crosses were flipped every 24-48h at 25ºC to synchronize the progeny and the wing phenotype was compared between adults or larvae of the same age. Genotypes are indicated in the figures legends.

**Wing imaginal discs dissection and whole-mount staining.** Late third instar larval wing imaginal disc were dissected in PBS at RT and then fixed with 4% PFA for 15 min at RT before being permeabilized with PBS/0.5% Triton-X100 for 5 min at RT, washed 3 times in PBS for 10 min at RT and directly processed for saturation using PBS/5% BSA for 1h at RT. To label the DNA the wing imaginal discs were incubated with Hoechst 33342 diluted 1/1000 in PBS for 5 min at RT then rinsed with MQ water and mounted in DaKo fluorescent mounting media. Genotypes are indicated in the figures legend.

**Quantification and statistical analysis**

**Densitometry on Western Blots.** Membranes were revealed using a Chemidoc imaging system (BioRad) and quantifications were performed using the Image Lab software (BioRad). To quantify and compare ubiquitination levels, results were expressed as ‘‘fold variation over Time 0 or over basal ubiquitination ’’ as they were normalized to the value at time 0 or in non-stimulation or co-expression conditions.

**Quantification of fluorescence intensity from confocal imaging.** Image analysis (cell segmentation and intensity measurements) was done using the open-source software Cell Profiler [[89](#_ENREF_89)]. The analysis pipeline is available on demand. Statistical analysis was done using GraphPadPrism software as indicated in legends to figures.

**Automated acquisition and analysis of cell images.** Samples were imaged on an automated ArrayScanVTI microscope (Thermo Scientific) using Zeiss 20x (NA 0.4) LD Plan-Neofluor air or Zeiss 40x (NA 0.75) EC Plan-Neofluar air objectives. Images were collected and analyzed with HCS studio v6.5.0 software. To enable direct comparison, all images within a given experiment were taken under the same magnification and exposure settings. Twelve to forty fields were systematically acquired for each fluorescent channel. Venus or EGFR positive spots and overlap with EEA1, Lamp1 or RAB4 markers were quantified using respectively: Compartmental analysis.V4 and Colocalization.V4 Bio-applications of HCS Studio software on 130 to 2000 individual cells. For Venus spots quantification, a primary object mask extracted from the Hoechst channel was created to count nuclei in each image. An enlarged circular mask derived from the primary mask was applied on the HA and Myc channels in order to filter out the negative thus non-transfected cells. A circular mask was then created in the Venus fluorescence channel in order to count Venus spots in transfected cells. Results correspond to the CircSpotCountCh2 feature from one representative experiment out of three.

For EGFR spot counting and co-localization quantification between Venus or EGFR spots with the various endosomal markers, an enlarged circular secondary mask derived from the nuclei primary mask was applied on each channel. The nuclear region was removed from the secondary mask. Spots corresponding either to Venus, EGFR or endosomal staining were identified, counted (extraction of CircSpotCountCh2 feature) and overlap area were quantified using the Target_I_and_II_OverlapArea parameter calculated on the bases of the overlap area between Venus spots or EGFR positive spots and the indicated endosomal marker. Statistical analysis was done using GraphPadPrism software as indicated in figure legends.

**Key ressources**

The following monoclonal (mAb) and polyclonal (pAb) primary antibodies were used in this study: mouse mAb against Flag-tag (M2) and rabbit pAb against USP8 (Sigma-Aldrich; resp. 1804 and HPA004869); mouse mAb against total ubiquitinated proteins (FK2), and rabbit pAb against Early Endosomal Antigen-1 (EEA1) (Enzo; resp. BML-PW8810 and ALX-210-239), rabbit pAb against GFP, rabbit pAb against CHMP1B (Abcam; ab 105706; Immunogen: Synthetic peptide corresponding to a region within N terminal amino acids 35-84 of human CHMP1B), rabbit pAb against IST1 and rabbit pAb against RAB4 (Abcam; resp. ab290, ab105706, ab139553 and ab13252), rat mAb against HA (Roche; 11867423001), mouse mAb against Myc (ATCC; hybridoma 9E10), rabbit mAb against Lamp1 (Cell Signaling; 9091), mouse mAb against EGFR (ThermoFisher; MA5-13070), rabbit mAb against K48 poly-ubiquitin chains (Millipore; clone Apu2), mouse mAb against extracellular domain of EGFR (ATCC; hybridoma Mab 108). The secondary antibodies HRP-coupled goat anti-mouse, rabbit and rat, and the TrueBlot anti-IgG of mice and rabbit are from Invitrogen. The secondary antibody Alexa 488 goat anti-mouse is from Life Technologies and the secondary antibodies goat anti-rat Cy3, goat anti-rabbit Cy3 and goat anti-mouse Cy5 are from Jackson ImmunoResearch. Human EGF recombinant protein was purchased from Sigma-Aldrich. IL-1β was purchased from R&D System.

**References**

89. Carpenter AE, Jones TR, Lamprecht MR, Clarke C, Kang IH, et al. (2006) CellProfiler: image analysis software for identifying and quantifying cell phenotypes. Genome Biol 7: R100.
